# Genetic diversity and stock identification of small abalone (*Haliotis diversicolor*) in Taiwan and Japan

**DOI:** 10.1371/journal.pone.0179818

**Published:** 2017-06-29

**Authors:** Te-Hua Hsu, Jin-Chywan Gwo

**Affiliations:** 1 Center of Excellence for the Oceans, National Taiwan Ocean University, Keelung, Taiwan; 2 Department of Aquaculture, National Taiwan Ocean University, Keelung, Taiwan; National Cheng Kung University, TAIWAN

## Abstract

Small abalone (*Haliotis diversicolor*) is a commercially valuable species for both fisheries and aquaculture. The production of annual farmed small abalone in Taiwan, once the highest in the world, has dramatically decreased in the past 15 years, and currently, the industry is close to collapse. Understanding the genetic diversity of small abalone and developing stock identification methods will be useful for genetic breeding, restoring collapsed stocks, managing stocks, and preventing illegal trade. We investigated 307 cultured and wild individuals from Taiwan, Japan, and Bali Island (Indonesia) by using the mitochondrial cytochrome c oxidase subunit I (COI) gene. Network analysis of mtDNA COI gene sequences revealed that the individuals collected from Taiwan, Japan, and Indonesia could be identified, and showed significant genetic divergence. In addition, the Indonesian population (*Haliotis diversicolor squamata*) was significantly different from the other populations and might need to be considered a separate species. We discovered a single nucleotide polymorphism marker in the mtDNA COI gene that can be used to distinguish the Taiwan population from the Japan population. We also developed a polymerase chain reaction-restriction fragment length polymorphism method for rapid detection. Furthermore, we could identify the cultured stocks, wild population, and hybrid stocks by using 6 microsatellites and amplified fragment length polymorphism. This study contributes useful tools for stock identification and the production of high-disease resistant small abalone strains (Japan × Taiwan or Taiwan × Japan). Efforts should be made to avoid unintentional random genetic mixing of the Taiwan population with the Japan population and subsequent breakdown of population differentiation, which impair local adaptation of the Taiwan wild population. Molecular markers revealed a split between the Taiwan and Japan populations, and the existence of a possible barrier to the free dispersal of small abalone is discussed.

## Introduction

The abalone genus *Haliotis* is the sole genus in the Haliotidae family, with approximately 56 species worldwide [1–2]. These species are widely distributed in temperate and tropical coastal reefs as well as rocky habitats, generally from sea level to a 30-m depth [3]. Approximately 14 abalone species are economically valuable for fishery production and aquaculture; most of them possess a large body and are distributed in temperate seas [4–5]. Small abalone is one of the small commercial abalone worldwide; it has a wide geographical distribution in warm water regions of East Asia, including Indonesia, the Philippines, Taiwan, the southern coastal waters of China, Jeju, Korea, and southern Japan (Kuroshio warm water region) [1–2,6].

In Taiwan, small abalone is commercially valuable for both fisheries and aquaculture because of its high price, rapid growth, and short life cycle [7–8]. Aquaculture of small abalone, invented by the Taiwanese, has rapidly expanded commercially owing to successful production of seeds and development of the unique land-based multiple-tier culture system in the 1980s [9–11]. In 2000, Taiwan produced the highest annual farmed small abalone yield, 2,497 tons, worldwide [12]. In the mid-1980s, Taiwanese small abalone farmers transplanted and exported broodstock and farming techniques to southern China (Fujian, Guangdong, Guangxi, and Hainan Provinces), and during the 1990s, the aquaculture industry spread and flourished rapidly in southern China coasts on a massive scale [8]. In 2001, the estimated small abalone output was approximately 3,878 tons in southern China [13–14]. In 2007, the total production of cultured abalone in China exceeded 20,000 tons, estimated to be worth CNY 50 billion (USD 8.2 million) annually, and nearly 60% of the output was produced on the coasts of southern China, particularly Fujian Province [8]. In 2004, approximately 60% of the abalone farms in China were land-based, but by 2010, a significant change was observed in this trend, and more than 95% of all farms were sea-based [8,15]. In 2013, China had more than 300 operating abalone farms, with the largest individual farm producing more than 90,694 tons annually, and either *H*. *discus hanni* or a hybrid between *H*. *discus hanni* and *H*. *discus discus* accounted for nearly 95% of abalone production [15].

However, disease outbreaks and substantial mortality of small abalone have occurred at the postlarval and grow-out stages in both Taiwan and southern China since 2002 [16]. In Taiwan, the production of small abalone has dramatically decreased in the past 15 years, and currently, the industry is close to collapse. Lack of suitable diatom feed for larvae, poor water quality, disease and infection, habitat degradation, and genetic problems are considered to be the causes of the dramatic decrease in small abalone production [8,17]. A series of investigations was conducted after a mortality event in Taiwan, and the official report published by the Taiwan Council of Agriculture indicated that no single causative factor contributed to the mortality event. The mortality event was often associated with the onset of winter and low water temperatures [17–19], which was possibly the final stressor that initiated the event.

Hatchery-reared stock has often been used as broodstock to produce the next generation for each year. Genetic degradation resulting from inbreeding may be the major reason for the mortality event, because high genetic diversity increases the population’s ability to withstand environmental perturbations and disease outbreaks [8,20–21]. Selection and hybridization are conventional strategies proven to be effective for the genetic improvement of mariculture mollusks, including small abalone [8,22–24]. Hybridization is an excellent method for increasing the growth rate and adaptability to environmental conditions. Intraspecies hybrids of small abalone between the Taiwan stock and the Japan wild population have shown positive hybrid vigors, such as disease resistance, higher survival rates and wider water temperature tolerance range, compared with the parental Taiwan stock [8,22]. Recently, the technique of crossbreeding female Taiwan stock and male Japan stock has been used widely in Taiwan and China.

Despite several decades of small abalone farming in Taiwan, the genetic structure of wild populations and cultured stocks of Taiwan small abalone remains unclear. In comparison with other abalone species, limited information is available on the genetic composition of *Haliotis diversicolor* endemic to Taiwan. The taxonomic status of small abalone is also controversial and unclear. Only minor morphological differences exist among *H*. *diversicolor*, *H*. *diversicolor aquatilis*, *H*. *diversicolor diversicolor*, and *H*. *diversicolor supertexta* regarding the relative ridge height of the shell [7]. Understanding the genetic diversity of small abalone and developing stock identification methods will be extremely useful for the ongoing breeding programs, future stock improvement, and conservation and management of this species.

Molecular genetics contributes to our understanding of the population, and this is particularly true for species that lack morphological differences among the population [25]. In this study, four types of genetic markers were applied to examine genetic diversity and detect any genetic difference among the small abalone taxa: amplified fragment length polymorphism (AFLP), microsatellite DNA (short sequence repeat, SSR), mtDNA cytochrome c oxidase subunit I (COI) gene, and single nucleotide polymorphism (SNP). First, we used DNA sequence variation in the mtDNA COI gene to elucidate phylogeny and test species delimitation of small abalone among the *H*. *diversicolor* complex (*H*. *diversicolor aquatilis*, *H*. *diversicolor diversicolor*, *H*. *diversicolor supertexta*, and *H*. *diversicolor squamata*) across most of its distribution throughout the biogeographical region. Furthermore, we used AFLP, SSR, and the mtDNA COI gene to examine the population genetic structure of wild, cultured, and intraspecific hybrid small abalone, *H*. *diversicolor*, with an emphasis on intensive sampling in Taiwan and the Japanese archipelago. Microsatellite loci and AFLP have been successfully used to determine population genetics and identify the hybrids of closely related species or sympatric species with similar morphology. Microsatellites have been used to detect loss of genetic diversity in cultured populations. Finally, we identified an SNP site in the mtDNA COI gene and used it to develop a polymerase chain reaction-restriction fragment length polymorphism (PCR-RFLP) method for identifying regional genetic differentiation between the Taiwan and Japan populations. In recent years, poaching, overfishing, climate warming and pollution have led to increasing concerns about the status of this abalone stock. Knowledge regarding the genetic diversity and the patterns of the stock structure is an essential for developing effective conservation approaches. However, no information is available on the genetic composition of wild and hatchery cultured small abalone in Taiwan. This information would be useful for the development of a more effective management for the small abalone industry and the conservation of small abalone, *H*. *diversicolor*, in Taiwanese waters.

## Materials and methods

### Ethics statement

This study was conducted in accordance with all Taiwan and Japan laws. Neither specific permits were required for the described field studies nor were specific permissions required for the locations/activities described in this study. The location is not privately owned or protected in any way. The field studies did not involve endangered or protected species.

### Sample collection and DNA extraction

**Fig 1:**
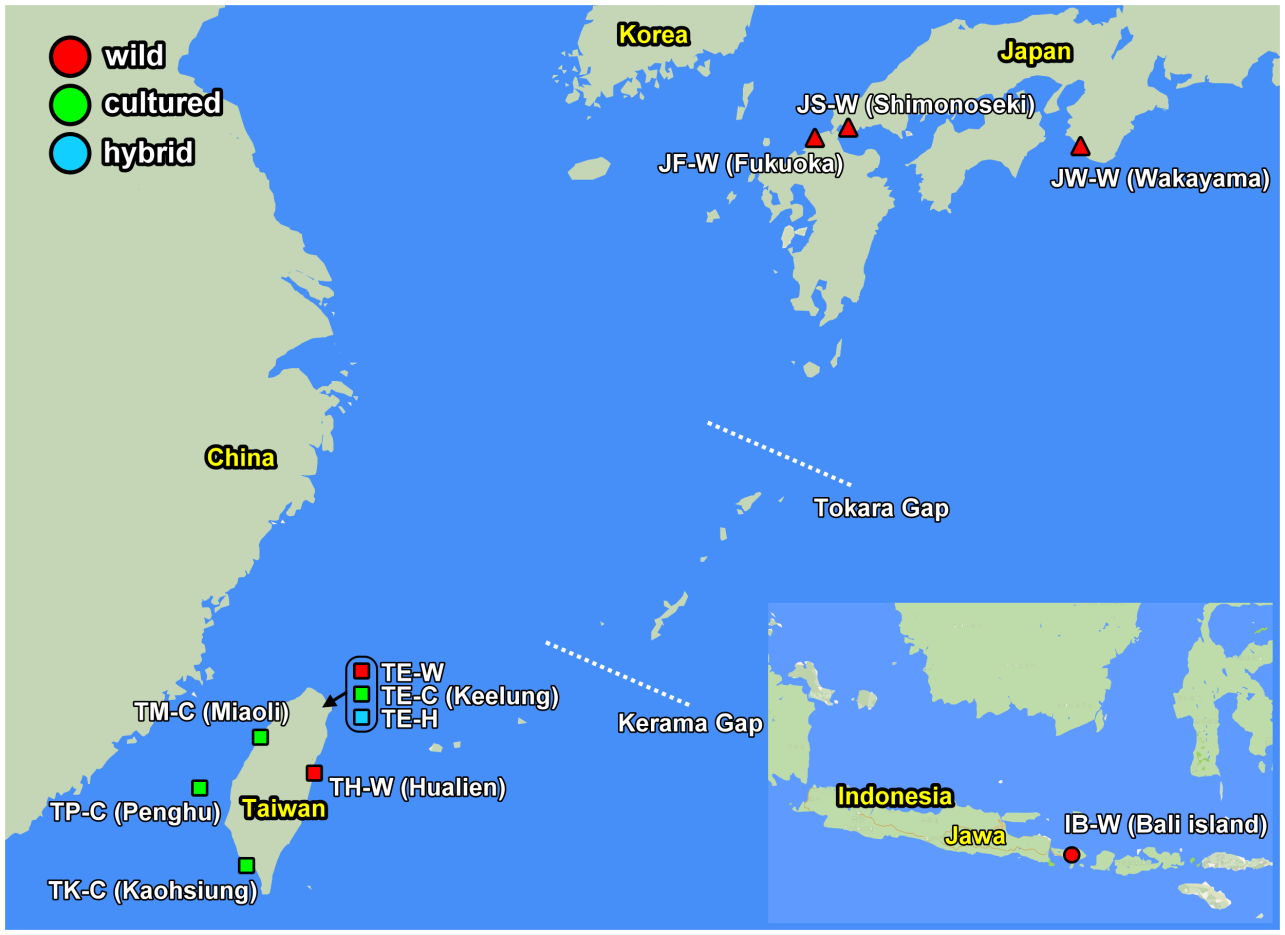
Map with an inset demonstrating the sampling locations of small abalone (*Haliotis diversicolor*) used in this study. JW-W, JF-W, and JS-W: wild population from Wakayama, Fukuoka, and Shimonoseki, Japan, respectively. IB-W: wild population from Bali, Indonesia. TP-C, TM-C, TE-C, TK-C, and TE-H: cultured stocks from Penghu Island, Miaoli, Keelung, Kaohsiung, and Keelung, Taiwan, respectively. H: intraspecies hybrid between female Taiwan cultured stock and male Japan wild population. TH-W and TE-W: wild population from Keelung and Hualien, Taiwan, respectively.

A total of 307 specimens of *H*. *diversicolor* and hybrids, including 7 individuals of *H*. *diversicolor squamata*, were used in this study (Table 1). Fresh specimens were obtained from 2007 to 2010 at 3 locations in Japan (JW-W: Wakayama-wild, JF-W: Fukuoka-wild, and JS-W: Shimonoseki-wild), 6 locations in Taiwan (TE-W: Keelung-wild, TH-W: Hualien-wild, TP-C: Penghu Island-cultured, TM-C: Miaoli-cultured, TE-C: Keelung-cultured, and TK-C: Kaohsiung-cultured), and one location in Indonesia (IB-W: Bali Island-wild) (Fig 1 and Table 1). One hybrid stock between TE-C (♀) and JW-W (♂) named TE-H was also obtained from the same hatchery as TE-C (Table 1). Fig 1 depicts the geographical positions of these populations, and Table 1 lists the sampling locations with the abbreviated population name and the sample size of each population. Small pieces of muscle tissues (about 3–5 mm) were prepared from fresh (use of 2% alcohol for anesthesia) or frozen samples and transported to the laboratory for molecular studies, and preserved in 95% ethanol. The standard proteinase K/phenol method was modified from an animal DNA extraction protocol. Moreover, 0.8% agarose gel electrophoresis was performed to assess DNA template quality.

**Table 1:** Sampling locations of 11 small abalone populations from Taiwan, Japan, and Indonesia.

Code	Sampling location	sources	*N*			SNP site (COI-116)	
			SSRs	AFLP	mtDNA(COI)	A	G
JW-WΔ	Wakayama, Japan	wild	8	5	22	18 (81.8%)	4 (18.2%)
JF-WΔ	Fukuoka, Japan	wild	12	10	32	27 (84.4%)	5 (15.6%)
JS-WΔ	Shimonoseki, Japan	wild	12	10	23	17 (73.9%)	6 (26.1%)
TE-W■	Keelung, Taiwan	wild	12	-	36	0 (0%)	36 (100%)
TH-W■	Hualien, Taiwan	wild	12	-	32	0 (0%)	32 (100%)
TP-C■	Penghu Island, Taiwan	cultured	10	-	27	0 (0%)	27 (100%)
TM-C■	Miaoli, Taiwan	cultured	10	-	29	0 (0%)	29 (100%)
TE-C■	Keelung, Taiwan	cultured	10	13	27	1 (4.7%)	26 (96.3%)
TK-C■	Kaohsiung, Taiwan	cultured	10	-	24	0 (0%)	24 (100%)
TE-H■	Keelung, Taiwan	hybrid*	-	10	48	0 (0%)	48 (100%)
Total	-	-	96	48	300	-	-
IB-W○	Bali Island, Indonesia	wild	-	-	7	-	-

Taiwan (■), Japan (Δ), and Indonesia (○). Refer to Table 1 and Fig 1 for population abbreviations.

### Mitochondrial DNA and analysis

The primer pair HdCOI, 5’-ATCTTCGGTATCTGATCTGGACTA-3’ and 5’-ACGGCGATGATTATTGTTGC-3’, was designed and used to amplify the partial mitochondrial COI gene, yielding an approximately 850-bp fragment. Amplifications of the COI gene as follows: 95°C for 5 min, 35 cycles of 95°C for 60 s, 58°C for 60 s, 72°C for 120 s. Moreover, DNA samples were purified using the QIAquick gel extraction kit (Qiagen, Taipei, Taiwan). Sequences were determined using the ABI 3770 (Applied Biosystems, Foster City, CA, USA) and edited and aligned in the DNA Baser (www.dnabaser.com). All sequences obtained in this study have been posted on GenBank (KX853541-KX853847).

All COI gene sequences obtained in this study, including those of the *H*. *diversicolor* complex, other *Haliotis* spp., and invertebrate taxa available from GenBank, were analyzed phylogenetically. The best-fitting nucleotide substitution model was tested by comparing the lowest Bayesian Information Criterion (BIC) scores by using MEGA v5 (S1 File) [26]. Bayesian phylogenetic analysis was performed under the best-fitting substitution model (HKY + I + G) by using BEAST v2.4.4 [27]. A strict clock model was inferred for each partition, representing the sites of first and second codons (one partition) and the third codons (second partition). The constant size coalescent process through time was assumed for the tree prior. Markov chains were run for 10,000,000 generations, with one tree saved every 1,000 generations. The program TRACER v1.5 was used to assess convergence diagnostics [28]. Support for each node was evaluated using posterior probabilities. Branch supports were also provided using 1,000 bootstrap replicates from neighbor joining, 500 replicates from maximum parsimony, and 100 replicates from maximum likelihood by using MEGA v5 [26].

A Bayesian species delimitation analysis of the general mixed Yule-coalescent model (GMYC) was used for species identification [29–30]. A data set containing multiple species/populations can be reconciled by using the branching rates in a phylogenetic tree, which correspond to divergence events between species (Yule process) or coalescent events between lineages within species (coalescent process) [29]. Both simulated and empirical studies for testing species delimitation under GMYC has been shown useful [29–30]. First, 100 trees were sampled from aforementioned phylogenetic analysis (the BEAST posterior distribution of trees under the same parameter and model). Markov chains were run for 10,000,000 generations, with sampling every 100,000 generations. Afterward, Bayesian Yule-coalescent model Markov chain Monte Carlo (GMYC-MCMC) analyses were assessed using the R package, bGMYC [30]. Each tree was run for 10,000 generations, sampled every 100 generations, and the first 1,000 generations were discarded as burn-in. The threshold parameter priors (t1 and t2) were set at 2 and 18, and the starting parameter value was set at 12.

Polymorphic sites, transitions, and transversions, were obtained using Arlequin v3.5 [31]. Number of haplotypes (*N*_hp_), average number of nucleotide differences (*k*), haplotype diversity (*h*), and nucleotide diversity (*π*) was calculated with DNASP v5.0 [32]. Mean number of pairwise distances (*p*-distance and Kimura 2-parameter model) were calculated by using MEGA v5 [26].

The significance of the population structure was tested using analysis of molecular variance (AMOVA) and pairwise *Φ*_ST_ values. Both statistical significances of the estimates were assessed by 10,000 permutations using Arlequin v3.5 [31]. The relationship among haplotypes was assessed using the median-joining network algorithm in the program Network 4.6.1.0 (Fluxus Engineering).

### PCR-RFLP (SNP site, COI-116)

The primer pair Hd116, 5’-TGGCGACGACCAATTATACA-3’ and 5’-GGGGTAGACTGTCCATCCTG-3’, was designed and used to amplify the partial mitochondrial COI gene including the SNP site (COI-116), yielding a 247-bp fragment. Amplifications of the SNP site (COI-116) were performed using an initial denaturation of 94°C for 3 min, followed by 35 cycles of 94°C for 60 s, 58°C for 30 s, and 72°C for 30 s and a final extension at 72°C for 3 min. Subsequently, the amplified samples (5 *μ*L) were subjected to endonuclease digestion by using the 4-base recognition enzymes MnlI (New England Bioladbs Inc., USA). Digestion was directly performed in the 10× NEB buffer 4 at 37°C for 3 h. The DNA fragments were separated on a 2.0% agarose gel with a fluorescent dye (EZ-vision N313, Amresco, USA).

### Microsatellites and analysis

A total of 6 microsatellite primer sets were used, with 5 from Ren et al. [33]: MS-1, MS-2, MS-3, MS-7, and MS-11, and one primer set (MS-19) from Zhan et al. [34]. Moreover, PCR amplification was performed in 20-*μ*L reaction volumes containing 5–10 ng template DNA; 1× PCR buffer (10 mM Tris and 50 mM KCl, pH 9.0); 200 μM of each dNTP, 1.5 mM MgCl_2_, and 0.5 U Taq polymerase (Promega, USA); and 4 pmol of each primer. Thereafter, PCR cycling was performed on an Autorisierter Thermocycler (Eppendorf, German) with initial denaturing at 95°C for 2 min, followed by 30 cycles of denaturing for 30 s at 95°C, annealing for 30 s at locus-specific temperatures, extension for 30 s at 72°C, and a final extension for 10 min at 72°C. The PCR products were denatured and visualized using denaturing polyacrylamide gels (6%), followed by silver staining [25]. Alleles were sized relative to a sequence ladder.

Procedures for microsatellite analysis were based on Hsu et al. [25]. The observed genetic diversity (*Ho*), expected genetic diversity (*He*), *F*_*IS*_ were calculated and the Chi-square Hardy–Weinberg equilibrium test were performed using GENALEX 6.41 [35]. To elucidate the population genetic structure from multilocus genotypes, the admixture model with correlated allele frequencies was performed using STRUCTURE v2.2 [36]. Three independent runs were performed for the total data set for K values ranging from 1 to 9. All runs were based on 100,000 iterations of burn-in followed by 500,000 iterations. The best estimation of the K value (number of groups) was conducted using STRUCTURE HARVESTER [37]. Summation and graphical representation of the STRUCTURE results were generated using CLUMPAK [38].

### AFLP and analysis

AFLP protocols were modified from Vos et al. [39] following protocols by Hsu et al. [25]. Genomic DNA for AFLP reactions were generated through restriction digestion, ligation, pre-amplification, and selective amplification. Genomic DNA was digested using EcoRI and Tru91 (Promega, USA). To generate DNA templates for pre-amplification and selective amplification, the digested DNA fragments were ligated using EcoRI and Msel adapters. Pre-amplification PCR reactions were performed using a pair of primers E-A/M-C. The pre-amplification product was diluted 50–100 fold with distilled water and was used as a template for subsequent selective PCR amplification. Selective amplification was performed using 6 pairs of primers: E-ATA/M-CGCA, E-AGC/M-CGCT, E-ACT/M-CGCA, E-AGG/M-CCTG, E-ACT/M-CGCG, and E-ACC/M-CCG. The PCR products were denatured and visualized using denaturing polyacrylamide gels (6%), followed by silver staining [25]. Band sizes were estimated using a standard AFLP DNA ladder and were analyzed using the imaging analyzing system (HP scanjet 5370c). The clear AFLP bands ranging from 100–300 bp were scored. Total bands, individual bands, polymorphic bands, private bands, and Nei’s standard genetic distance (*D*) were calculated using the Dice similarity coefficient [40] and Excel VBA [41]. Genetic diversity (*He*) was calculated according to the allelic frequencies (square root method) by using GenAlEx 6.41 [35]. Principal coordinate analysis (PCoA) was performed using a matrix of squared Euclidean distances computed from individual binary data by using GenAlEx 6.41 [35].

## Results

### Mitochondrial DNA

#### Phylogenetic analysis and species delimitation

**Fig 2:**
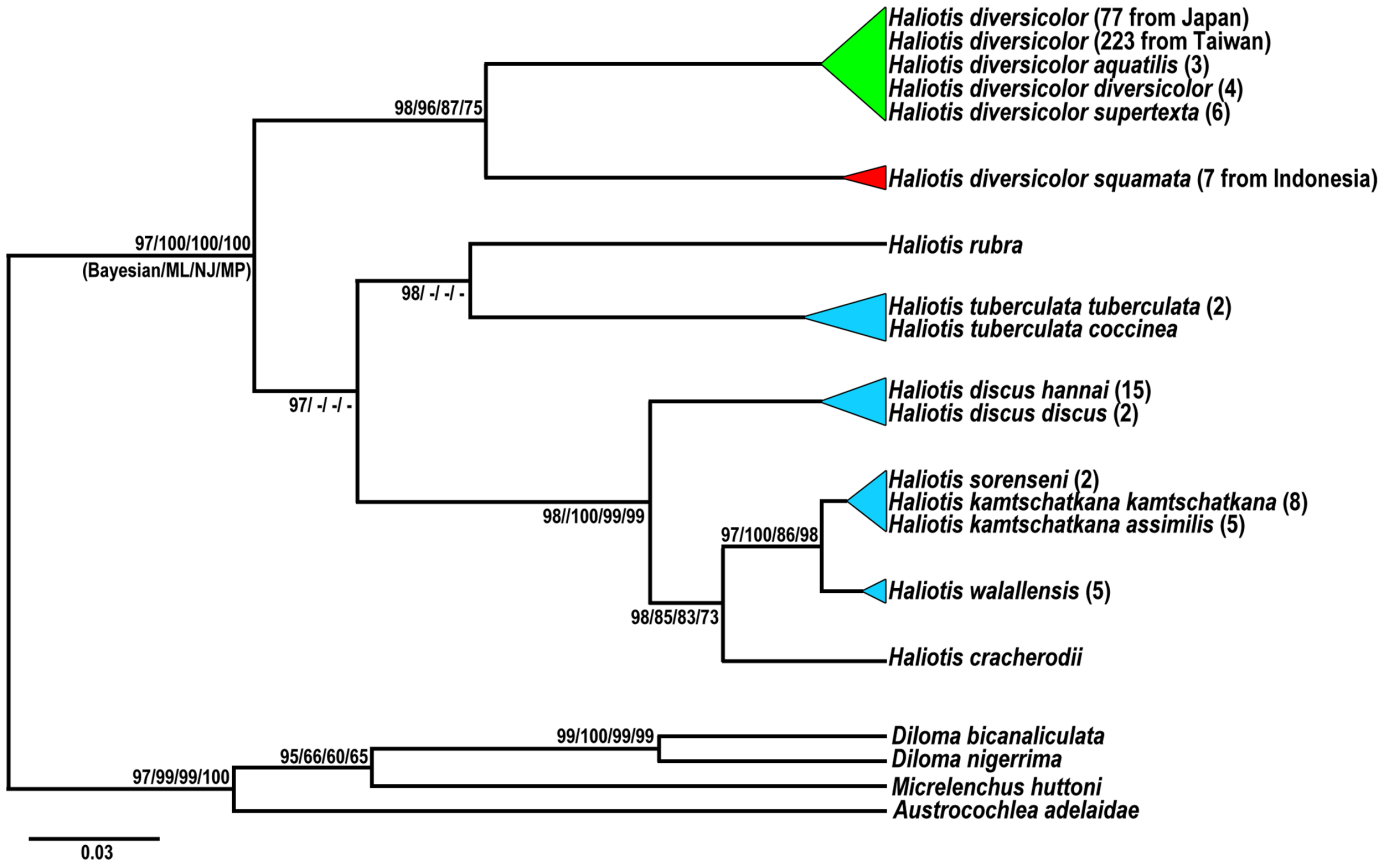
Bayesian inference majority-rule consensus topology derived from the partial COI gene of the *Haliotis* genus. Numbers at nodes indicate topological support: posterior probabilities in Bayesian analysis, 1,000 bootstrap replicates from neighbor joining (NJ), and 100 bootstrap replicates from maximum likelihood (ML) (Bayesian-NJ-ML).

**Fig 3:**
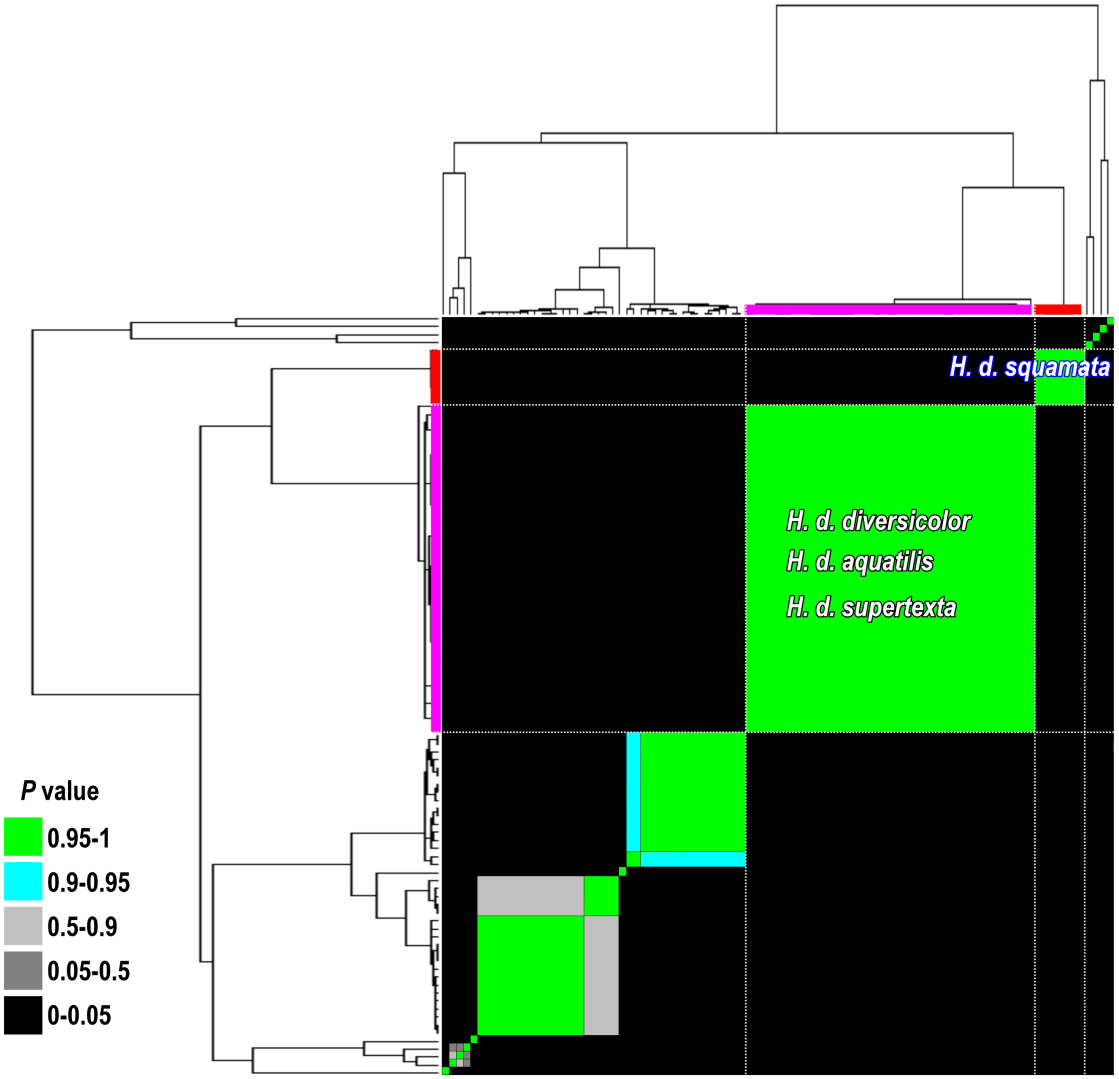
Likelihood and Bayesian analyses for species delimitation tested under the general mixed Yule-coalescent model (GMYC). The phylogenetic tree is the maximum clade credibility tree from BEAST (same topology as Fig 3A). The colored table is an individual-by-individual matrix. Cells are colored according to the posterior probability that the corresponding individuals are conspecific. The *P* values (0–0.05), represented as black cells, indicate individuals that are not conspecific.

Phylogenetic analysis was performed using partial mitochondrial COI gene sequences (808 bp) from 307 individuals of small abalone, the *H*. *diversicolor* complex, including samples collected from 3 locations in Japan, 7 locations in Taiwan, and one location in Indonesia (Fig 1 and Table 1). In addition, phylogenetic analysis was performed for 13 *Haliotis* taxa (species or subspecies) from GenBank (S1 Table). Eight clades were present in the Bayesian phylogenetic analysis, as follows: *H*. *aliotis rubra*, *H*. *cracherodii*, *H*. *discus* (*H*. *discus discus* and *H*. *discus hannai*), *H*. *walallensis*, *H*. *kamtschatkana* complex (*H*. *kamtschatkana assimilis*, *H*. *kamtschatkana kamtschatkana*, and *H*. *sorenseni*), *H*. *tuberculata* (*H*. *tuberculata tuberculata* and *H*. *tuberculata coccinea*), *H*. *diversicolor* complex (*H*. *diversicolor diversicolor*, *H*. *diversicolor supertexta*, and *H*. *diversicolor aquatilis*), and *H*. *diversicolor squamata* (Fig 2). The COI sequences of these 8 clades revealed high genetic variation (*p*-distance, 0.017–0.194) and represented monophyletic relationships. The lowest p-distance of 0.017 was detected between *H*. *walallensis* and the *H*. *kamtschatkana* complex, and the highest *p*-distance of 0.194 was detected between *H*. *cracherodii* and the *H*. *diversicolor* complex (S2 Table). The p-distance between the *H*. *diversicolor* complex and *H*. *diversicolor squamata* was 0.135 (S2 Table). The species delimitation test under the GMYC also suggested that the *H*. *diversicolor* complex and *H*. *diversicolor squamata* are different species (Fig 3).

#### Genetic diversity

Partial mitochondrial COI gene sequences (808 bp) were obtained from 300 individuals of small abalone, *H*. *diversicolor* complex, collected from 3 locations in Japan and 7 locations in Taiwan (Fig 1 and Table 1). Only one of the 94 polymorphic sites (1.16%) corresponded to nonsynonymous substitutions. A total of 94 haplotypes were obtained from all sequences, of which 73 were private haplotypes (77.66%) (Table 2). The number of haplotypes (*N*_hp_) per location ranged between 6 in TK-C and 25 in JF-W. The number of private haplotypes per location ranged between 0 in TK-C and TP-C and 20 in JF-W (Table 2). Moreover, the Japan stocks (JW-W, JF-W, and JS-W) showed the highest number of haplotypes (*N*_hp_) and the highest number of private haplotypes. H_1 and H_10 were the most common haplotypes in the Japan stocks, and H_4 and H_30 were the most common haplotypes in the Taiwan stocks (see S3 Table).

**Table 2:** Sampling sites, number of sequences (*n*), number of halpotypes (*N*_hp_), number of private haplotypes (*N*p), average number of nucleotide differences (*k*), haplotype diversity (*h*), nucleotide diversity (*π*) for each clade of *Haliotis diversicolor* collected from the coastal waters of Japan and Taiwan.

		Genetic diversity				
code	*n*	*N*_hp_	*N*_p_ (%)	*K*	*h*	*π* (%)
JW-W	22	17	12 (54.5)	4.27 ± 2.20	0.97 ± 0.03	0.53 ± 0.30
JF-W	32	25	20 (62.5)	3.44 ± 1.81	0.97 ± 0.02	0.43 ± 0.25
JS-W	23	17	14 (60.9)	4.15 ± 2.14	0.96 ± 0.02	0.51 ± 0.30
TE-W	36	21	10 (27.8)	3.21 ± 1.70	0.94 ± 0.03	0.40 ± 0.23
TH-W	32	14	02 (06.3)	3.14 ± 1.67	0.89 ± 0.04	0.39 ± 0.23
TP-C	27	8	00 (00.0)	1.99 ± 1.16	0.84 ± 0.05	0.25 ± 0.16
TM-C	29	12	04 (13.8)	3.13 ± 1.67	0.90 ± 0.03	0.39 ± 0.23
TE-C	27	10	03 (11.1)	2.96 ± 1.60	0.86 ± 0.04	0.37 ± 0.22
TK-C	24	6	00 (00.0)	1.37 ± 0.87	0.70 ± 0.08	0.17 ± 0.12
TE-H	48	20	08 (16.7)	3.45 ± 1.80	0.93 ± 0.02	0.43 ± 0.25

The average number of nucleotide differences (*k*), haplotype diversity (*h*), and nucleotide diversity (*π*) was high, indicating no inbreeding in the wild populations from Taiwan and Japan, and a slight inbreeding in cultured stocks from Taiwan (Table 2). Wild populations from Japan (JF-W, JF-W and JS-W) had a higher average number of nucleotide differences (*k* = 3.442–4.273), haplotype diversity (*h* = 0.964–0.974), and nucleotide diversity (*π* = 0.426–0.529). By contrast, cultured stock from Taiwan (TP-C and TK-C) had a lower average number of nucleotide differences (*k* = 1.366–1.991), haplotype diversity (h = 0.703–0.838), and nucleotide diversity (*π* = 0.169–0.247) (Table 2).

#### Population differentiation

**Fig 4:**
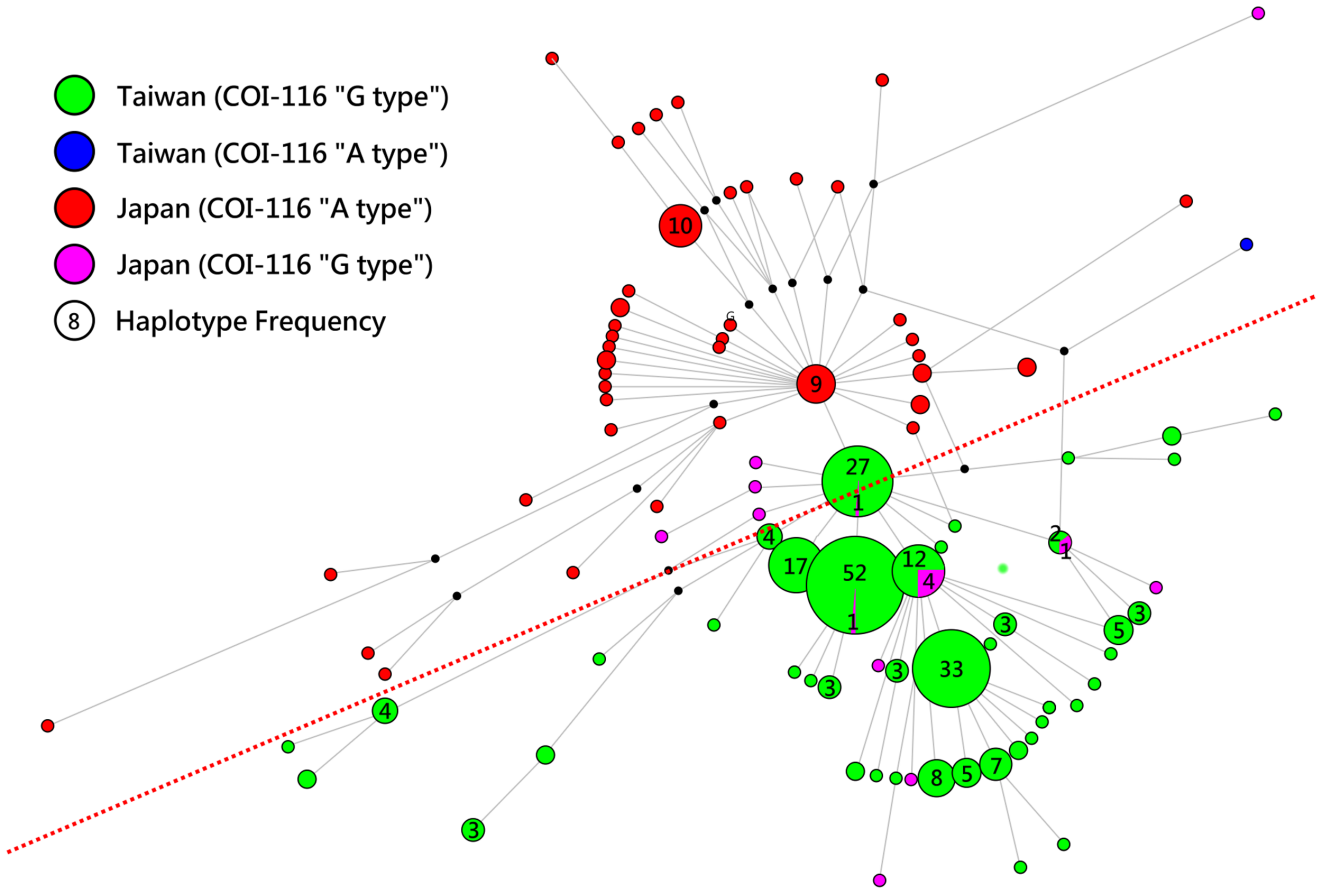
Haplotype network for 300 haplotypes of small abalone (*Haliotis diversicolor*). Green, blue, red, and pink circles indicate haplotypes among sampling locations. The size of the circles is proportional to the haplotype frequencies. Lengths of the line are relative to the number of mutations among haplotypes. Haplotype networks represent the COI gene and states of the SNP site (COI-116) for each haplotype. Each circle corresponds to a distinct haplotype and lines connecting haplotypes to one mutational step. The numbers indicate additional haplotypes. Two subclades (Taiwan and Japan) are separated by a thin dashed line.

Two levels of genetic division were obtained for the *Haliotis* samples collected from Taiwan, Japan, and Bali Island (Indonesia). The first level, two distinct clades (*H*. *diversicolor* complex and *H*. *diversicolor squamata*) according to the Bayesian phylogenetic tree (Fig 2), was not considered to be the same species for population differentiation. Second, the haplotype network revealed restriction of gene flow between the *Haliotis* samples collected from Taiwan and Japan (Fig 4). Pairwise *Φ*_ST_ values were 0.044–0.159 and supported significant differentiation (*P* = 0–0.001) between the Taiwan stocks and Japan wild populations (Table 3). Pairwise *Φ*_ST_ values were 0.005–0.068 and supported no significant differentiation (*P* = 0.034–0.269) between the Taiwan cultured stocks and Taiwan wild populations, except for TE-W and TK-C (Table 2). No significant differentiation was observed within the Taiwan wild populations (*Φ*_ST_ = -0.003, *P* = 0.507) and the Japan wild populations (*Φ*_ST_ = -0.002–0.011, *P* = 0.113–0.493). Limited differentiation or no significant differentiation was observed among the Taiwan cultured stocks (TP-C, TM-C, TE-C, TK-C, and TE-H) (Table 3).

**Table 3:** Pairwise *Φ*_*ST*_ values (below diagonal) and associated *P* values (above diagonal) based on mtDNA COI sequences between populations of *Haliotis diversicolor* collected from Japan and Taiwan.

	JW-W	JF-W	JS-W	TE-W	TH-W	TP-C	TM-C	TE-C	TK-C	TE-H
JW-W	—	0.113^NS^	0.237^NS^	0.001**	0***	0***	0***	0***	0***	0.001**
JF-W	0.011	—	0.493^NS^	0***	0***	0***	0***	0***	0***	0***
JS-W	0.006	-0.002	—	0.001**	0***	0***	0***	0***	0***	0***
TE-W	0.047	0.04	0.051	—	0.507^NS^	0.261^NS^	0.034*	0.208^NS^	0.005*	0.198^NS^
TH-W	0.07	0.055	0.061	-0.003	—	0.189^NS^	0.038^NS^	0.185^NS^	0.040 ^NS^	0.269^NS^
TP-C	0.094	0.085	0.087	0.006	0.012	—	0.024*	0.133^NS^	0.005*	0.070^NS^
TM-C	0.068	0.061	0.07	0.025	0.03	0.041	—	0.008*	0***	0.016*
TE-C	0.077	0.075	0.088	0.009	0.012	0.022	0.05	—	0.075 ^NS^	0.458^NS^
TK-C	0.159	0.14	0.148	0.068	0.042	0.097	0.128	0.036	—	0.002*
TE-H	0.047	0.044	0.052	0.006	0.005	0.021	0.028	-0.001	0.069	—

*0.05 ≥ *P* ≥ 0.01;

**0.01 > *P* ≥ 0.001;

***P < 0.001;

^NS^, not significant.

#### PCR-RFLP (SNP site, COI-116)

**Fig 5:**
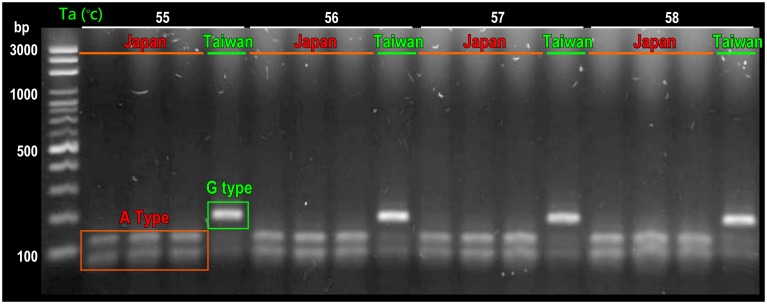
Electrophoresis result of the PCR-RFLP method for rapid detection of the SNP site (COI-116). The “G type” showed one band and mainly found in the Taiwan populations; “A type” showed two bands and mainly observed in the Japan populations. Annealing temperatures (Ta) are 55–58°C.

We found a SNP site, COI-116, in the mtDNA COI gene sequences among *Haliotis* samples collected from Taiwan and Japan. The “G type” and “A type” were mainly found in the Taiwan and Japan populations, respectively (Fig 4 and Table 1). The “G type” accounted for 100% of the Taiwan wild populations and culture stocks, except for TE-C (96.3%). The “A type” accounted for 73.9%-84.4% of the Japan wild populations. Furthermore, the PCR-RFLP method was developed successfully for rapid detection (Fig 5).

### Microsatellites

**Fig 6:**
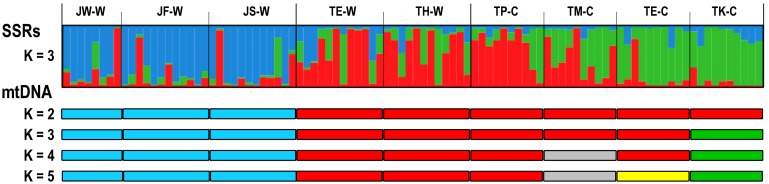
Structure analysis of small abalone populations. Estimated population structure based on the highest probability STRUCTURE run at K = 3 by using SSRs data. Each individual is represented by a thin vertical line, which is partitioned into K colored segments that represent the individual’s estimated membership fractions in each of the K clusters. Cluster 1 = blue, cluster 2 = red, cluster 3 = green. Estimated population structure based on mtDNA COI sequences by using the highest probability SAMOVA (*F*_*CT*_) run from K = 2 to K = 5 (S6 Table). JW-W, JF-W, and JS-W: wild population from Wakayama, Fukuoka, and Shimonoseki, Japan, respectively. TP-C, TM-C, TE-C, and TK-C: cultured stocks from Penghu Island, Miaoli, Keelung, and Kaohsiung, Taiwan, respectively. TH-W and TE-W: wild population from Keelung and Hualien, Taiwan, respectively.

Six microsatellites were used to verify the population genetic structure of 3 Japan wild stocks (JW-W, JF-W, and JS-W), 2 Taiwan wild stocks (TE-W and TH-W), and 4 Taiwan cultured stocks (TP-C, TM-C, TE-C, and TK-C). S4 Table presents the summary statistics for genetic variation at the 6 microsatellite loci in 9 populations. In a Bayesian analysis of the population structure of each stock, the number of clusters should be 3, at which a high Lnp (x/k) with little variance is obtained (data not shown). Fig 6 displays the clustering results with k = 3. Cluster 1 (blue) corresponds to the Japan wild stocks, and cluster 2 (red) and cluster 3 (green) correspond to the Taiwan stocks (Fig 6). The genetic characteristics of cultured stocks are different from those of wild stocks. All individuals from wild stocks are mainly admixtures of 3 clusters (JW-W, JF-W, JS-W, TE-W, and TH-W). The Taiwan cultured stocks (TM-C, TE-C, and TK-C) are dominant in cluster 3 (green), except for the TP-C stock from Penghu Island. A high level of admixture of wild and cultured individuals is suggested for the TP-C stock. These results are consistent with the pairwise *Φ*_ST_ and *F*_*ST*_ analysis of population structure in Table 3 and S5 Table.

### AFLP profiles

Hybrid identification was performed using 6 different AFLP primer combinations. The AFLP profiles proved to be highly reproducible. No difference was observed among the replicates, although different amounts of DNA were used. For the TE-H cross, the 6 AFLP primer combinations generated a total of 149 markers, of which 71 were polymorphic, with an average of 47.65 markers per primer set (S7 Table). All 6 primer combinations produced a high percentage of polymorphic bands, with the average being 73.96% (S7 Table). Region-specific bands from both the wild Japan population and the Taiwan cultured stock coexisted in all 48 progenies of the TE-H cross. This finding indicated the presence of both parental genomes in the hybrids and confirmed that the hybrids are the offspring of 2 genetically combined parents.

**Fig 7:**
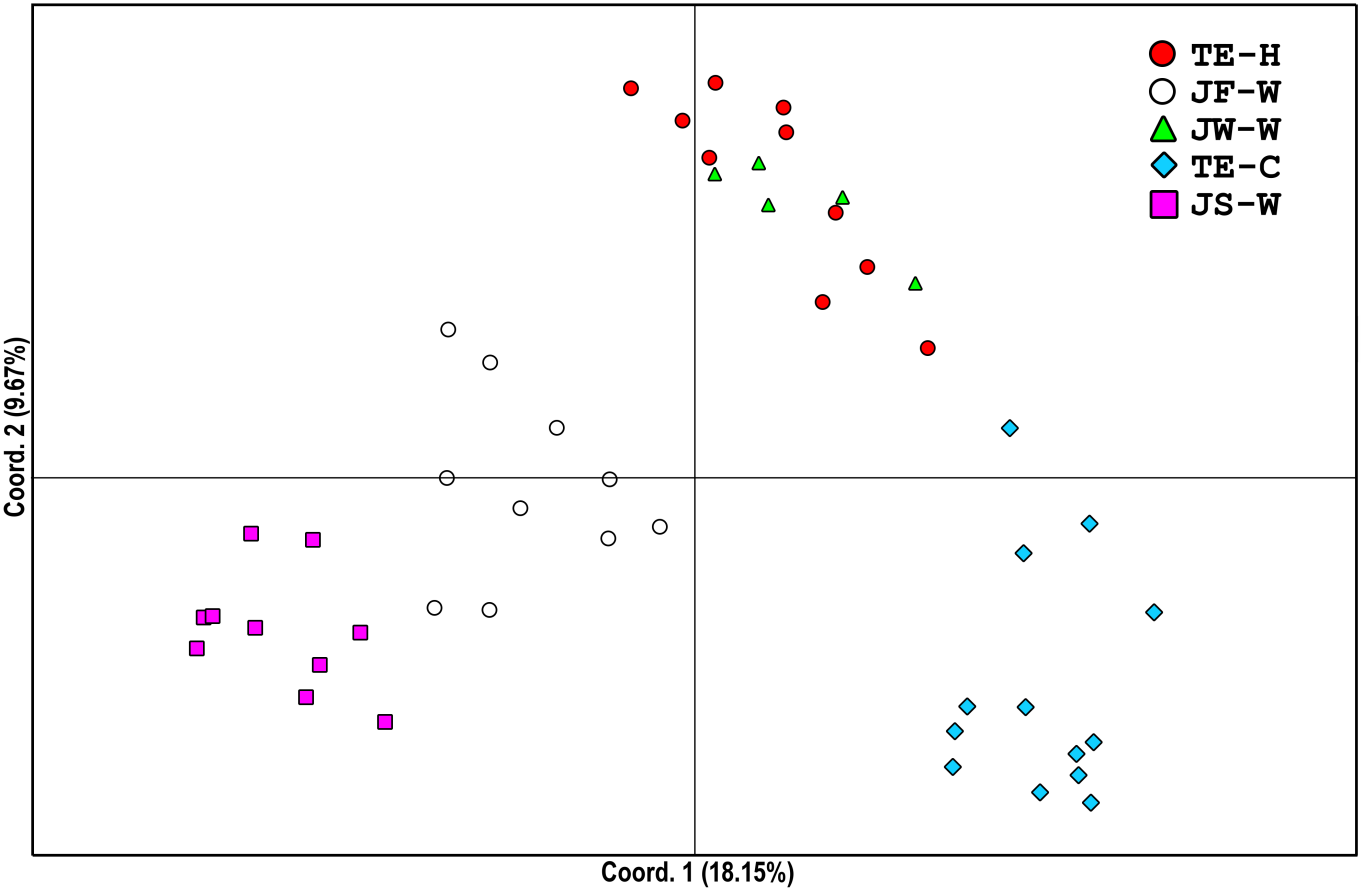
Principal coordinates analysis (PCoA) of small abalone populations. Plot of the first and second principal coordinates of AFLP genotypes for small abalone stocks based on a similarity matrix using PCoA. The first and second coordinates account for 18.15% and 9.67% of the total variation, respectively. JW-W, JF-W, and JS-W: wild population from Wakayama, Fukuoka, and Shimonoseki, Japan, respectively. TE-C: cultured stocks from Keelung, Taiwan. TE-H: intraspecific hybrid between female Taiwan cultured stock and male Japan wild population.

Fig 7 illustrates the results of principal component analysis. In Fig 7, scatter plots of the AFLP data on the first principal coordinate and the second coordinate present each wild population and cultured stock separately. All individuals of different stocks were concentrated in one large cluster. Most individuals of the same stocks were clustered together, although some individuals from TE-H showed larger genetic distances from the main cluster. The profiles of the wild Japan populations (JS-W, JF-W, and JW-W) and the Taiwan cultured stock (TE-C) indicated that they were relatively diverse, and region-specific bands were present in each population or stock (data not shown). Intraspecific hybrids of TE-H showed intermediate patterns between their parents.

## Discussion

Molecular markers have proven to be a powerful tool for taxonomic identification and genetic relationship determination for species with ambiguous or cryptic morphological characteristics [42–44]. An et al. [45] used 7 RAPD markers to distinguish 6 different species of Pacific abalone at the molecular level and effectively classify them into species-specific DNA fingerprint groups. The taxonomic classification of *H*. *discus* is also unclear, and discriminating these species according to morphological characteristics is difficult. Six microsatellite markers proved to be successful for evaluating the significant genetic differences between 2 subspecies, *H*. *discus hannai* and *H*. *discus discus* [46]. Our phylogenetic results (Figs 2 and 3) are consistent with the previous observations made using mitochondrial DNA (16S rRNA, COI) and nuclear DNA (ITS-1, 18S rRNA) [42,47]. In this study, we found that all 4 haplotypes, *H*. *diversicolor*, *H*. *diversicolor aquatilis*, *H*. *diversicolor diversicolor*, and *H*. *diversicolor supertexta*, were clustered together. No divergence was detected among these haplotypes, suggesting that they are the same species. Hence, *H*. *diversicolor*, *H*. *diversicolor aquatilis*, *H*. *diversicolor diversicolor*, and *H*. *diversicolor supertexta* are not detectable for stock management. *H*. *diversicolor squamata*, an Indonesian small abalone, is significantly different from the other 4 small abalone species (p-distance = 0.135) and may need to be considered a separate species (Figs 2 and 3 and S7 Table). Samples of small abalone species collected from additional locations, such as China, Vietnam, and the Philippines, may provide further insight into the population genetic structure of the *H*. *diversicolor* complex.

Small abalone (*Haliotis diversicolor*) is a small, worldwide commercial abalone and has a wide geographical distribution in the warm water regions of the northwest Pacific (Indonesia, the Philippines, Taiwan, southern China, southern Korea, and southern Japan) [1–2,6]. The mitochondrial COI gene haplotype network revealed significant genetic differentiation and restriction of gene flow between small abalone samples collected from Taiwan and Japan (Fig 4). The STRUCTURE analysis, which provides patterns of microsatellite divergence, of the Taiwan and Japan wild populations also indicated that small abalone populations around Kuroshio Current are genetically grouped and may have developed local adaptations to their particular habitats (Fig 6). Two Taiwan wild populations (TE-W and TH-W) showed markedly different patterns compared with the other 3 Japan wild populations (Fig 6). Both the mitochondrial COI gene haplotype and microsatellite pattern results indicate that genetic divergence may have occurred between the 2 geographical populations (i.e., Taiwan and Japan). Small abalone (*Haliotis diversicolor*) is small and has a maximum shell length of approximately 10 cm; it mainly inhabits shallow subtidal areas in subtropical and temperate zones of the southern area of mainland Japan, except for Okinawa, where small abalone was introduced from Taiwan and Japan [48]. The small abalone shows limited dispersal behavior, which is attributed to their benthic habit with weak migratory ability after settlement, following a brief free-swimming stage that is complete within a few days [48]. The relatively short planktonic larvae phase implies that small abalone cannot disperse far, unlike other nonsessile marine animals, such as fishes. The Kuroshio Current, which has an average speed of 1 ms^−1^ [49], may play a decisive role in separating small abalone populations between Okinawa and mainland Japan, because the pelagic larvae of small abalone, with restricted migratory ability, are unlikely to cross the Kuroshio Current.

During the Pleistocene, land bridges, lower sea levels, and current hydrodynamic regimes profoundly changed in the northwest Pacific [50–52]. These dramatic changes in environmental and geographical regimes are a potential mechanism that affected the population genetic structure of marine organisms. The Tokara Gap and the Kerama Gap, which separate the Japanese and Ryukyu Arc into 3 parts (Fig 1), are very deep, indicating that land bridges probably never existed between the respective islands in the glacial period [50–51]. Therefore, these gaps are assumed to have interfered with genetic relationships among many animals. The 2 deep gaps have been suggested to play crucial roles in the formation of the existing biogeographic features in this region on the basis of phylogeographic patterns in terrestrial animals, amphibians, and reptiles or freshwater organisms easily influenced by segregation among islands [50–51]. Kojima et al. [51] demonstrated the significant effects of these 2 gaps on the establishment of genetic compositions of the intertidal gastropod genus Cerithidea. However, the hypotheses proposed to explain the phylogeography of current terrestrial animals between Taiwan and the Ryukyu Islands might not be adequate for marine animals, such as neon damsel fish (*Pomacentrus coelestis*) [52] and red seabream (*Pagrus major*) [53]. The strong Kuroshio Current is likely to contribute to gene flow and genetic homogeneity among the red sea bream populations along the Pacific coast of Japan [53]. The Tokara Gap has been considered to be a major barrier for some organisms migrant with the Kuroshio Current, including ayu (*Plecoglossus altivelis*), red seabream, and neon damsel fish [52–54]. Liu et al. [52] revealed that the Tokara Gap, but not the Kerama Gap, affected the population genetic structure of *P*. *coelestis* between mainland Japan and Okinawa. Based on the disjunctive distribution of small abalone species coupled with the genetic differences between the Taiwan and Japan wild populations observed in this study, small abalone is likely formed by several small breeding units, implying high genetic variability. The biogeographic pattern of small abalone is analogous with that of marine reef fish P. coelestis, amphidromous fish ayu, and oceanodromous demersal fish red seabream. The Tokara Gap and the Kerama Gap might also act as barriers to the free dispersal of small abalone.

Genetic diversity, indicated by haplotype diversity (*h*), was found to be very high for both the Taiwan and Japan wild populations of small abalone (Table 2). The values generated in the present study are comparable with those derived from the mtDNA and nuclear DNA markers for other abalone species [43,55–56]. In contrast to previous study findings [57], we found no evidence for mtDNA differentiation, geographical isolation, and inbreeding in the Taiwan wild populations of small abalone. A high level of genetic diversity in the mtDNA COI region may be attributed to the reasonable large population number and high nucleotide mutation rates [58]. Similar high genetic variation has been reported for Korea and China small abalone [34,43], indicating that this observation may be a common characteristic of this species. No marked decrease was identified in the genetic diversity (0.703–0.897) of the Taiwan cultured stocks (TP-C, TM-C, TE-C, and TK-C) (Table 2).

It has been reported that aquaculture practices tend to reduce genetic variation in cultured stocks of abalone species [59–60]. Genetic analysis of various hatchery fish species has revealed similar phenomena [59,61]. Theoretically, the wild population provides all genetic variability to each separate cultured stock. With no migration occurring between different cultured stocks, the decline of genetic variability in cultured stocks over several generations is normally regarded as the result from interacting bottleneck, drift, and artificial and natural selection in the closed culturing systems [59,61–62]. Loss of genetic variation in the hatchery cultured stocks of *H*. *diversicolor* has been reported in China by using AFLP [52]. An et al. [43] developed 20 novel polymorphic MS primer sets for small abalone, *H*. *diversicolor*, by using 454 GS-FLX pyrosequencing, and they surveyed genetic diversity at these loci in the wild and hatchery cultured populations of this species from Jeju, Korea. They emphasized that although no significant reductions were found in genetic diversity between the hatchery cultured stock and the wild population of small abalone, the genetic diversity parameters showed that the hatchery cultured seeds had different genetic compositions. The mtDNA genetic diversity (h) of all 4 Taiwan cultured stocks (TP-C, TK-C, TM-C, and TE-C) was not significantly different from that of the Taiwan wild population (TH-W) (Table 3). By contrast, the other 2 Taiwan cultured stocks (TK-C and TM-C) were significantly different from the Taiwan wild population (TE-W). Little differentiation or no significant differentiation was also observed among the Taiwan cultured stocks (TP-C, TM-C, TE-C, TK-C, and TE-H) (Tables 2 and 3). Because the TE-H and TE-C stocks shared the same female broodstocks, their pairwise ΦST value was very high (0.458; Table 3). In the present study, high mtDNA haplotype diversity (h) was maintained, but the nuclear genetic structures (composition) of the Taiwan cultured stocks were substantially altered compared with those of the Taiwan wild populations (Fig 6 and Table 2). Loss of nuclear diversity (*π*) without an accompanying loss of mtDNA haplotype diversity (h) may be explained by the loss of low-frequency alleles, which usually occurs during a population bottleneck. Continued intensive breeding practices lead to reduced genetic diversity if the size of effective populations is limited. The slight reduction of genetic diversity in the Taiwan cultured stocks (TP-C, TM-C, and TE-C) compared with that in the Taiwan wild population (TE-W and TH-W) may be attributed to the small number of parent abalone used for reproduction, because the cultured stocks consistently underwent a population bottleneck and subsequent genetic drift. The hatchery cultured TK-C stock showed marked reduction in the number of haplotypes (*N*_hp_ = 6) and haplotype diversity (*h* = 0.703) in comparison with the Taiwan wild populations and other cultured stocks. This finding is consistent with the observation that the cultured stocks obtained without exchanging broodstocks are characterized by reduced genetic variation due to genetic drift in the aquaculture environment.

The Japan wild populations (JW-W, JF-W, and JS-W) exhibited the highest number of haplotypes (*N*_hp_) and the highest number of private haplotypes (Table 2). The number of haplotypes (*N*_hp_) per location ranged between 6 in the Taiwan cultured stock (TK-C) and 25 in the Japan wild population (JF-W). The number of private haplotypes per location ranged between 0 in the Taiwan cultured stock (TK-C and TP-C) and 20 in the Japan wild population (JF-W) (Table 2). The loss of low-frequency or rare alleles from hatchery cultured stocks may be a more relevant measure of genetic variation than heterozygosity. The loss of alleles exerts more damaging effects than the change in allele frequencies, because allele frequencies can be changed by genetic drift, but recovering a lost allele is impossible. The total extinction of any allele can be considered more harmful than a decrease in overall heterozygosity [61].

Pairwise *Φ*_ST_ values, AMOVA, and AFLP all supported significant differentiation between cultured stocks and wild populations (Figs 2 and 4, Tables 2 and 3). Such significant differentiation between cultured stocks and wild populations has also been reported for many other abalone species, such as *H*. *kamtschatkana*, *H*. *discus hannai*, *H*. *rubra*, and *H*. *midae* [59,61–62]. This significant differentiation results from the bottleneck (founder effect), artificial selection, and drift. Notably, we did not discover the signals of differentiation among the 3 Japan wild populations and 2 Taiwan wild populations, suggesting high gene flow in these populations. Compared with the Taiwan wild populations, the Taiwan cultured stocks exhibited a significantly smaller maximal shell length and a significantly higher growth coefficient [11]. In Taiwan, the market size of small abalone is approximately 5 cm. Because the cultured stock reaches the market size much more rapidly than the wild populations do, the small abalone industry of Taiwan selects broodstocks according to their reproductive performance (success), and not body size. Several other genetic characters (i.e., disease resistance and stress tolerance) may be a tradeoff over several or numerous generations in a closed cultured environment. Intraspecies hybrids of small abalone between the Taiwan stock and Japan wild population have shown positive heterosis (hybrid vigors), such as disease resistance and wider temperature tolerance range, compared with the parental Taiwan stock [8,22]. Because private abalone famers imported wild stocks from Japan in a desultory fashion, the technique of crossbreeding between female Taiwan stock and male Japan stock has been used widely in Taiwan and China since 2013. However, in Taiwan, the situation of small abalone (*Haliotis diversicolor*) aquaculture is in disarray. The precise origin and pedigree information of broodstocks are unknown, and the genetic management of small abalone is difficult.

The release of cultured seeds into natural habitats is one measure for restoring collapsed abalone stocks. Stocking hatchery-reared organisms in wild populations usually greatly affect the genetic integrity of native wild populations and can even dilute and/or replace native wild populations when seeds are released in numbers that exceed the carrying capacity of release regions [63]. Hamasaki and Kitada [64] suggested that “the abalone stock should be managed according to each fishing ground by considering the status of abalone recruitment and abundance of prey in algal forests; hatchery-reared juveniles should be released where the algal forests are abundant but where abalone recruitment is limited”. The larvae of small abalone species selectively settle on crustose coralline algae (CCA) and grow on CCA for at least a few months after settlement [48,65–67]. A storm event caused by a typhoon appears to be an indispensable cue to trigger the synchronous spawning of small abalone (*H*. *diversicolor*) [65–66]. In northeastern Taiwan, farmers have built small abalone ponds at the intertidal zone and the littoral zone to culture small abalone. These ponds are mostly adjacent to the sea; thus, pond water freely exchanges with open sea water [68–69]. Typhoons and storms frequently occur in Taiwan, with an annual average frequency of 3–4 [70]. Farmed marine fishes were spawning in sea-cages and releasing huge amount of fertilized eggs that survived and dispersed into surrounding sea waters. Because farmed fish experience severe artificial selection during their larval stage and also through tough selective breeding for optimal aquaculture stocks. These practices had changed the genetic composition of farmed versus wild fish, which also altered the fitness of wild fish through interbreeding between farm escapees and wild fish [71]. The “escape” of fertilized eggs and larvae from small abalone farms may lead to undesirable genetic effects in native wild populations through interbreeding, as well as ecological effects through predation, competition, displacement, and the transfer of parasites or/and diseases to the native wild population.

In conclusion, no information has previously been available on the genetic structure and variation between and within the cultured stocks and wild populations of small abalone in Taiwan, until now. This study showed no genetic differentiation between the 2 wild populations (TE-W and TH-W) and revealed significant differentiation among the cultured stocks (TP-C, TM-C, TE-C, and TK-C). The genetic differences between geographical populations (Taiwan and Japan) can provide wide genetic diversity for selective breeding and hybridization for the sustainable use of this species. To avoid potentially harmful disease spread and the ecological and genetic consequences of escape through spawning, the pond water used for culturing small abalone in Taiwan according to the present coastal farming style should be treated properly before discharge. Efforts should also be made to avoid the unintentional genetic mixing of the Taiwan native wild population with the Japan population and subsequent breakdown of population differentiation, which impair local adaptation of the Taiwan native wild population. Continued monitoring of the genetic diversity among wild populations, cultured stocks, and intraspecies hybrids by using molecular markers is important for effective management of the small abalone industry and conservation of small abalone in Taiwanese waters.

## Supporting information

S1 Table *Haliotis* taxa from GenBank used in phylogenetic analysis. (DOCX)

S2 Table Pairwise genetic differences (*p*-distance) among *Haliotis* taxa based on mtDNA COI sequences. (DOCX)

S3 Table Haplotype frequencies of small abalone (*Haliotis diversicolor*) per population. Numbers in bold are private haplotypes.
(DOCX)

S4 Table Summary statistics for genetic variation at 6 microsatellite loci in 9 populations of small abalone. (DOCX)

S5 Table Pairwise *F*_*ST*_ values (below diagonal) and associated *P* values (above diagonal) based on SSRs data between populations of *Haliotis diversicolor* collected from Japan and Taiwan. (DOCX)

S6 Table Hierarchical analysis of molecular variance (AMOVA) of small abalone populations based on mtDNA COI sequences collected from Japan and Taiwan by using SMOVA. (DOCX)

S7 Table Summary of AFLP results obtained by using 6 primers in 6 populations of small abalone (*Haliotis diversicolor*). *D*: genetic distance (based on similarity coefficient); Shannon Information Index (*I*); *He*: expected heterozygosity (based on allele frequency); U*He*: unbiased expected heterozygosity.
(DOCX)

S1 File Model test results. (PDF)
