# Supplementary material for: Genetic diversity and stock identification of small abalone (Haliotis diversicolor) in Taiwan and Japan
Source: PLoS One. 2017 Jun 29;12(6):e0179818. doi: 10.1371/journal.pone.0179818 (PMC5491045; doi:10.1371/journal.pone.0179818)
Supplement: S1 Table — (DOCX) [file pone.0179818.s001.docx]

**S1 Table.** ***Haliotis* taxa from GenBank used in phylogenetic analysis.**

| Species (sample size) | Accession numbers |
| --- | --- |
| *Haliotis diversicolor* |  |
| *Haliotis diversicolor diversicolor* (5) | EU244364, AY146402, HM595786, AY319441, AY319440 |
| *Haliotis diversicolor supertexta* (6) | AY319443, HQ832672, HQ832671, AY146401, AY319442,  HQ832673 |
| *Haliotis diversicolor aquatilis* (3) | AB236705, AB236707, AB236706 |
| *Haliotis rubra* | AY588938 |
| *Haliotis tuberculata* |  |
| *Haliotis tuberculata tuberculata* (2) | FJ599667, FJ605488 |
| *Haliotis tuberculata coccinea* | FJ605486 |
| *Haliotis kamtschatkana* |  |
| *Haliotis kamtschatkana assimilis* (5) | JF285142, JF285150, JF285145, JF285146, JF285147 |
| *Haliotis kamtschatkana kamtschatkana* (8) | JF285135, JF285137, JF285139, JF285138, JF285132,  JF285133, JF285134, JF285136 |
| *Haliotis sorenseni* (2) | JF285152, JF285153 |
| *Haliotis walallensis* (5) | JF285122, JF285123, JF285124, JF285126, JF285127 |
| *Haliotis discus* |  |
| *Haliotis discus discus* (2) | AY319446, AY319447 |
| *Haliotis discus hannai* (16) | JF748799, AY319444, JF748784, JF748787, JF748790,  JF748793, JF748802, JF748803, AY319445, JF748825,  JF748828, JF748815, KF724723, JF748812, EU595789 |
| *Haliotis cracherodii* | JF285162 |
| *Diloma bicanaliculata* | AY858093 |
| *Diloma nigerrima* | AY858094 |
| *Austrocochlea adelaidae* | AY858081 |
| *Micrelenchus huttoni* | AY858079 |
