# Supplementary material for: Genetic diversity and stock identification of small abalone (Haliotis diversicolor) in Taiwan and Japan
Source: PLoS One. 2017 Jun 29;12(6):e0179818. doi: 10.1371/journal.pone.0179818 (PMC5491045; doi:10.1371/journal.pone.0179818)
Supplement: S2 Table — (DOCX) [file pone.0179818.s002.docx]

**S2 Table.** **Pairwise genetic differences (*p*-distance) among *Haliotis* taxa based on mtDNA COI sequences.**

|  | R | T | K | W | D | C | O | I |
| --- | --- | --- | --- | --- | --- | --- | --- | --- |
| R | - |  |  |  |  |  |  |  |
| T | 0.145 | - |  |  |  |  |  |  |
| K | 0.157 | 0.167 | - |  |  |  |  |  |
| W | 0.165 | 0.166 | 0.017 | - |  |  |  |  |
| D | 0.171 | 0.175 | 0.088 | 0.086 | - |  |  |  |
| C | 0.176 | 0.180 | 0.066 | 0.066 | 0.095 | - |  |  |
| O | 0.158 | 0.170 | 0.174 | 0.177 | 0.186 | 0.194 | - |  |
| I | 0.168 | 0.178 | 0.172 | 0.173 | 0.185 | 0.174 | 0.135 | - |

R: *Haliotis rubra* (*n* = 1)*,* T: *Haliotis tuberculata* (*n* = 3), K: *Haliotis kamtschatkana* complex (*n* = 15), W: *Haliotis walallensis* (*n* = 5), D: *Haliotis discus* (*n* = 17), C: *Haliotis cracherodii* (*n* = 1), O: *Haliotis diversicolor* complex (*n* = 314, Taiwan and Japan), I: *Haliotis diversicolor squamata* (*n* = 7, Indonesia).
