# Supplementary material for: Genetic diversity and stock identification of small abalone (Haliotis diversicolor) in Taiwan and Japan
Source: PLoS One. 2017 Jun 29;12(6):e0179818. doi: 10.1371/journal.pone.0179818 (PMC5491045; doi:10.1371/journal.pone.0179818)
Supplement: S3 Table — Numbers in bold are private haplotypes. (DOCX) [file pone.0179818.s003.docx]

**S3 Table.** **Haplotype frequencies of small abalone (*Haliotis diversicolor*) per populatio**n. Numbers in bold are private haplotypes.

|  | JW-W | JF-W | JS-W | TE-W | TH-W | TP-C | TM-C | TE-C | TK-C | TE-H |
| --- | --- | --- | --- | --- | --- | --- | --- | --- | --- | --- |
| H_1 | 0.182 | 0.063 | 0.130 | 0 | 0 | 0 | 0 | 0 | 0 | 0 |
| H_2 | **0.045** | 0 | 0 | 0 | 0 | 0 | 0 | 0 | 0 | 0 |
| H_3 | 0.045 | 0.031 | 0 | 0 | 0 | 0 | 0 | 0 | 0 | 0 |
| H_4 | 0.045 | 0 | 0 | 0.083 | 0.031 | 0.148 | 0.034 | 0.259 | 0.208 | 0.125 |
| H_5 | **0.091** | 0 | 0 | 0 | 0 | 0 | 0 | 0 | 0 | 0 |
| H_6 | **0.045** | 0 | 0 | 0 | 0 | 0 | 0 | 0 | 0 | 0 |
| H_7 | **0.045** | 0 | 0 | 0 | 0 | 0 | 0 | 0 | 0 | 0 |
| H_8 | **0.045** | 0 | 0 | 0 | 0 | 0 | 0 | 0 | 0 | 0 |
| H_9 | **0.045** | 0 | 0 | 0 | 0 | 0 | 0 | 0 | 0 | 0 |
| H_10 | 0.045 | 0.156 | 0.130 | 0 | 0 | 0 | 0 | 0 | 0 | 0 |
| H_11 | **0.045** | 0 | 0 | 0 | 0 | 0 | 0 | 0 | 0 | 0 |
| H_12 | **0.045** | 0 | 0 | 0 | 0 | 0 | 0 | 0 | 0 | 0 |
| H_13 | **0.045** | 0 | 0 | 0 | 0 | 0 | 0 | 0 | 0 | 0 |
| H_14 | **0.091** | 0 | 0 | 0 | 0 | 0 | 0 | 0 | 0 | 0 |
| H_15 | **0.045** | 0 | 0 | 0 | 0 | 0 | 0 | 0 | 0 | 0 |
| H_16 | 0.045 | 0 | 0 | 0 | 0 | 0 | 0 | 0 | 0 | 0.042 |
| H_17 | **0.045** | 0 | 0 | 0 | 0 | 0 | 0 | 0 | 0 | 0 |
| H_18 | 0 | **0.063** | 0 | 0 | 0 | 0 | 0 | 0 | 0 | 0 |
| H_19 | 0 | **0.031** | 0 | 0 | 0 | 0 | 0 | 0 | 0 | 0 |
| H_20 | 0 | 0.031 | 0.130 | 0.083 | 0 | 0.111 | 0.138 | 0 | 0 | 0.021 |
| H_21 | 0 | **0.031** | 0 | 0 | 0 | 0 | 0 | 0 | 0 | 0 |
| H_22 | 0 | **0.031** | 0 | 0 | 0 | 0 | 0 | 0 | 0 | 0 |
| H_23 | 0 | **0.031** | 0 | 0 | 0 | 0 | 0 | 0 | 0 | 0 |
| H_24 | 0 | **0.031** | 0 | 0 | 0 | 0 | 0 | 0 | 0 | 0 |
| H_25 | 0 | **0.031** | 0 | 0 | 0 | 0 | 0 | 0 | 0 | 0 |
| H_26 | 0 | **0.031** | 0 | 0 | 0 | 0 | 0 | 0 | 0 | 0 |
| H_27 | 0 | **0.031** | 0 | 0 | 0 | 0 | 0 | 0 | 0 | 0 |
| H_28 | 0 | **0.063** | 0 | 0 | 0 | 0 | 0 | 0 | 0 | 0 |
| H_29 | 0 | **0.031** | 0 | 0 | 0 | 0 | 0 | 0 | 0 | 0 |
| H_30 | 0 | 0.031 | 0 | 0.194 | 0.281 | 0.185 | 0.138 | 0.259 | 0.500 | 0.167 |
| H_31 | 0 | **0.031** | 0 | 0 | 0 | 0 | 0 | 0 | 0 | 0 |
| H_32 | 0 | **0.031** | 0 | 0 | 0 | 0 | 0 | 0 | 0 | 0 |
| H_33 | 0 | **0.031** | 0 | 0 | 0 | 0 | 0 | 0 | 0 | 0 |
| H_34 | 0 | **0.031** | 0 | 0 | 0 | 0 | 0 | 0 | 0 | 0 |
| H_35 | 0 | **0.031** | 0 | 0 | 0 | 0 | 0 | 0 | 0 | 0 |
| H_36 | 0 | **0.031** | 0 | 0 | 0 | 0 | 0 | 0 | 0 | 0 |
| H_37 | 0 | **0.031** | 0 | 0 | 0 | 0 | 0 | 0 | 0 | 0 |
| H_38 | 0 | **0.031** | 0 | 0 | 0 | 0 | 0 | 0 | 0 | 0 |
| H_39 | 0 | **0.031** | 0 | 0 | 0 | 0 | 0 | 0 | 0 | 0 |
| H_40 | 0 | 0 | **0.043** | 0 | 0 | 0 | 0 | 0 | 0 | 0 |
| H_41 | 0 | 0 | **0.043** | 0 | 0 | 0 | 0 | 0 | 0 | 0 |
| H_42 | 0 | 0 | **0.043** | 0 | 0 | 0 | 0 | 0 | 0 | 0 |
| H_43 | 0 | 0 | **0.043** | 0 | 0 | 0 | 0 | 0 | 0 | 0 |
| H_44 | 0 | 0 | **0.043** | 0 | 0 | 0 | 0 | 0 | 0 | 0 |
| H_45 | 0 | 0 | **0.043** | 0 | 0 | 0 | 0 | 0 | 0 | 0 |
| H_46 | 0 | 0 | **0.043** | 0 | 0 | 0 | 0 | 0 | 0 | 0 |
| H_47 | 0 | 0 | **0.043** | 0 | 0 | 0 | 0 | 0 | 0 | 0 |
| H_48 | 0 | 0 | **0.043** | 0 | 0 | 0 | 0 | 0 | 0 | 0 |
| H_49 | 0 | 0 | **0.043** | 0 | 0 | 0 | 0 | 0 | 0 | 0 |
| H_50 | 0 | 0 | **0.043** | 0 | 0 | 0 | 0 | 0 | 0 | 0 |
| H_51 | 0 | 0 | **0.043** | 0 | 0 | 0 | 0 | 0 | 0 | 0 |
| H_52 | 0 | 0 | **0.043** | 0 | 0 | 0 | 0 | 0 | 0 | 0 |
| H_53 | 0 | 0 | **0.043** | 0 | 0 | 0 | 0 | 0 | 0 | 0 |
| H_54 | 0 | 0 | 0 | 0.167 | 0.156 | 0.333 | 0.172 | 0.111 | 0 | 0.104 |
| H_55 | 0 | 0 | 0 | **0.056** | 0 | 0 | 0 | 0 | 0 | 0 |
| H_56 | 0 | 0 | 0 | 0.056 | 0 | 0.037 | 0.034 | 0 | 0.042 | 0 |
| H_57 | 0 | 0 | 0 | 0.028 | 0.031 | 0 | 0 | 0 | 0 | 0.042 |
| H_58 | 0 | 0 | 0 | 0.028 | 0.031 | 0 | 0 | 0 | 0 | 0 |
| H_59 | 0 | 0 | 0 | 0.028 | 0.063 | 0 | 0 | 0.037 | 0 | 0 |

**S3 Table** continued

|  | JW-W | JF-W | JS-W | TE-W | TH-W | TP-C | TM-C | TE-C | TK-C | TE-H |
| --- | --- | --- | --- | --- | --- | --- | --- | --- | --- | --- |
| H_60 | 0 | 0 | 0 | **0.028** | 0 | 0 | 0 | 0 | 0 | 0 |
| H_61 | 0 | 0 | 0 | **0.028** | 0 | 0 | 0 | 0 | 0 | 0 |
| H_62 | 0 | 0 | 0 | **0.028** | 0 | 0 | 0 | 0 | 0 | 0 |
| H_63 | 0 | 0 | 0 | **0.028** | 0 | 0 | 0 | 0 | 0 | 0 |
| H_64 | 0 | 0 | 0 | **0.028** | 0 | 0 | 0 | 0 | 0 | 0 |
| H_65 | 0 | 0 | 0 | 0.028 | 0 | 0 | 0 | 0 | 0.042 | 0 |
| H_66 | 0 | 0 | 0 | **0.028** | 0 | 0 | 0 | 0 | 0 | 0 |
| H_67 | 0 | 0 | 0 | **0.028** | 0 | 0 | 0 | 0 | 0 | 0 |
| H_68 | 0 | 0 | 0 | **0.028** | 0 | 0 | 0 | 0 | 0 | 0 |
| H_69 | 0 | 0 | 0 | 0.028 | 0 | 0.074 | 0 | 0 | 0 | 0 |
| H_70 | 0 | 0 | 0 | 0.028 | 0.031 | 0 | 0 | 0 | 0 | 0.021 |
| H_71 | 0 | 0 | 0 | 0.028 | 0.031 | 0 | 0 | 0.074 | 0 | 0.021 |
| H_72 | 0 | 0 | 0 | **0.028** | 0 | 0 | 0 | 0 | 0 | 0 |
| H_73 | 0 | 0 | 0 | 0 | 0.063 | 0 | 0 | 0 | 0 | 0.021 |
| H_74 | 0 | 0 | 0 | 0 | 0.063 | 0 | 0.103 | 0.037 | 0 | 0.042 |
| H_75 | 0 | 0 | 0 | 0 | **0.031** | 0 | 0 | 0 | 0 | 0 |
| H_76 | 0 | 0 | 0 | 0 | 0.063 | 0.074 | 0.069 | 0.111 | 0.042 | 0.146 |
| H_77 | 0 | 0 | 0 | 0 | **0.031** | 0 | 0 | 0 | 0 | 0 |
| H_78 | 0 | 0 | 0 | 0 | 0 | 0.037 | 0.034 | 0 | 0 | 0 |
| H_79 | 0 | 0 | 0 | 0 | 0 | 0 | **0.241** | 0 | 0 | 0 |
| H_80 | 0 | 0 | 0 | 0 | 0 | 0 | **0.034** | 0 | 0 | 0 |
| H_81 | 0 | 0 | 0 | 0 | 0 | 0 | **0.034** | 0 | 0 | 0 |
| H_82 | 0 | 0 | 0 | 0 | 0 | 0 | **0.034** | 0 | 0 | 0 |
| H_83 | 0 | 0 | 0 | 0 | 0 | 0 | 0.069 | 0 | 0 | 0.021 |
| H_84 | 0 | 0 | 0 | 0 | 0 | 0 | 0 | **0.037** | 0 | 0 |
| H_85 | 0 | 0 | 0 | 0 | 0 | 0 | 0 | **0.037** | 0 | 0 |
| H_86 | 0 | 0 | 0 | 0 | 0 | 0 | 0 | **0.037** | 0 | 0 |
| H_87 | 0 | 0 | 0 | 0 | 0 | 0 | 0 | 0 | 0 | **0.021** |
| H_88 | 0 | 0 | 0 | 0 | 0 | 0 | 0 | 0 | 0 | **0.021** |
| H_89 | 0 | 0 | 0 | 0 | 0 | 0 | 0 | 0 | 0 | **0.063** |
| H_90 | 0 | 0 | 0 | 0 | 0 | 0 | 0 | 0 | 0 | **0.021** |
| H_91 | 0 | 0 | 0 | 0 | 0 | 0 | 0 | 0 | 0 | **0.021** |
| H_92 | 0 | 0 | 0 | 0 | 0 | 0 | 0 | 0 | 0 | **0.042** |
| H_93 | 0 | 0 | 0 | 0 | 0 | 0 | 0 | 0 | 0 | **0.021** |
| H_94 | 0 | 0 | 0 | 0 | 0 | 0 | 0 | 0 | 0 | **0.021** |
