# Supplementary material for: Genetic diversity and stock identification of small abalone (Haliotis diversicolor) in Taiwan and Japan
Source: PLoS One. 2017 Jun 29;12(6):e0179818. doi: 10.1371/journal.pone.0179818 (PMC5491045; doi:10.1371/journal.pone.0179818)
Supplement: S4 Table — (DOCX) [file pone.0179818.s004.docx]

**S4 Table.** **Summary statistics for genetic variation at 6 microsatellite loci in 9 populations of small abalone.**

| Population (*N*) | | MS-1 | MS-2 | MS-3 | MS-7 | MS-11 | MS-19 | Average |
| --- | --- | --- | --- | --- | --- | --- | --- | --- |
| JW-W (8) | *A* | 5 | 3 | 4 | 3 | 6 | 7 | 4.67 |
|  | Ar | 4.850 | 2.875 | 3.867 | 3.000 | 5.733 | 6.733 | 4.51 |
|  | *H*_o_ | 0.500 | 0.375 | 1.000 | 0.286 | 0.375 | 0.375 | 0.485 |
|  | *H*_e_ | 0.675 | 0.542 | 0.675 | 0.473 | 0.808 | 0.883 | 0.676 |
|  | *F*_IS_ | 0.273 | 0.323 | -0.534 | 0.415 | 0.553* | 0.592* | 0.297* |
| JF-W (12) | *A* | 4 | 6 | 8 | 3 | 10 | 9 | 6.67 |
|  | Ar | 3.004 | 5.139 | 6.754 | 2.877 | 8.266 | 7.298 | 5.56 |
|  | *H*_o_ | 0.333 | 0.750 | 0.917 | 0.091 | 0.333 | 0.667 | 0.515 |
|  | *H*_e_ | 0.308 | 0.688 | 0.870 | 0.498 | 0.913 | 0.880 | 0.693 |
|  | *F*_IS_ | -0.086 | -0.094 | -0.057 | 0.825* | 0.645* | 0.251 | 0.265* |
| JS-W (12) | *A* | 5 | 6 | 8 | 2 | 8 | 7 | 6.00 |
|  | Ar | 3.691 | 5.192 | 6.291 | 1.980 | 7.130 | 6.412 | 5.12 |
|  | *H*_o_ | 0.167 | 0.833 | 0.667 | 0.167 | 0.091 | 0.500 | 0.404 |
|  | *H*_e_ | 0.435 | 0.746 | 0.848 | 0.290 | 0.887 | 0.859 | 0.678 |
|  | *F*_IS_ | 0.627* | -0.122 | 0.221 | 0.436 | 0.902* | 0.429* | 0.415* |
| TE-W (12) | *A* | 3 | 5 | 9 | 3 | 7 | 13 | 6.67 |
|  | Ar | 2.941 | 4.565 | 6.589 | 2.577 | 5.781 | 9.709 | 5.36 |
|  | *H*_o_ | 0.333 | 0.667 | 0.583 | 0.333 | 0.333 | 1.000 | 0.542 |
|  | *H*_e_ | 0.620 | 0.790 | 0.808 | 0.409 | 0.804 | 0.942 | 0.729 |
|  | *F*_IS_ | 0.473 | 0.162* | 0.287* | 0.193 | 0.596* | -0.065 | 0.265* |
| TH-W (12) | *A* | 3 | 6 | 7 | 2 | 5 | 12 | 5.83 |
|  | Ar | 2.878 | 5.042 | 5.272 | 1.980 | 4.842 | 8.776 | 4.80 |
|  | *H*_o_ | 0.364 | 0.417 | 0.750 | 0.167 | 0.500 | 0.667 | 0.477 |
|  | *H*_e_ | 0.537 | 0.743 | 0.736 | 0.290 | 0.772 | 0.917 | 0.666 |
|  | *F*_IS_ | 0.333 | 0.450* | -0.021 | 0.436 | 0.362* | 0.282 | 0.292* |
| TP-C (10) | *A* | 2 | 5 | 5 | 1 | 6 | 8 | 4.50 |
|  | Ar | 1.997 | 4.665 | 4.100 | 1.000 | 5.303 | 6.700 | 3.96 |
|  | *H*_o_ | 0.400 | 0.800 | 0.800 | 0.000 | 0.300 | 0.900 | 0.640 |
|  | *H*_e_ | 0.337 | 0.768 | 0.600 | 0.000 | 0.789 | 0.837 | 0.666 |
|  | *F*_IS_ | -0.200 | -0.043 | -0.358 | NA | 0.633* | -0.080 | 0.042* |
| TM-C (10) | *A* | 2 | 7 | 6 | 1 | 7 | 7 | 5.00 |
|  | Ar | 1.982 | 6.285 | 5.524 | 1.000 | 6.384 | 6.018 | 4.53 |
|  | *H*_o_ | 0.300 | 0.300 | 0.900 | 0.000 | 0.600 | 0.800 | 0.580 |
|  | *H*_e_ | 0.268 | 0.858 | 0.811 | 0.000 | 0.847 | 0.832 | 0.723 |
|  | *F*_IS_ | -0.125 | 0.663* | -0.117 | NA | 0.303* | 0.040 | 0.207* |
| TE-C (10) | *A* | 4 | 5 | 4 | 2 | 7 | 9 | 5.17 |
|  | Ar | 3.682 | 4.399 | 3.400 | 1.700 | 6.367 | 8.093 | 4.61 |
|  | *H*_o_ | 0.600 | 0.800 | 0.500 | 0.100 | 0.500 | 0.900 | 0.567 |
|  | *H*_e_ | 0.642 | 0.747 | 0.574 | 0.100 | 0.816 | 0.921 | 0.633 |
|  | *F*_IS_ | 0.069 | -0.075 | 0.135 | 0.000 | 0.400 | 0.024 | 0.110 |
| TK-C (10) | *A* | 5 | 6 | 7 | 3 | 7 | 10 | 6.33 |
|  | Ar | 4.400 | 5.318 | 5.704 | 3.000 | 6.236 | 8.246 | 5.48 |
|  | *H*_o_ | 0.600 | 0.700 | 0.700 | 0.600 | 0.200 | 0.900 | 0.617 |
|  | *H*_e_ | 0.758 | 0.805 | 0.689 | 0.700 | 0.847 | 0.889 | 0.782 |
|  | *F*_IS_ | 0.217* | 0.137 | -0.016 | 0.150 | 0.774* | -0.013 | 0.220* |

*N* = number of samples, *A* = allele number, Ar = allele richness, *H*_e_ = expected heterozygosity, *H*_o_ = observed heterozygosity, *F*_IS_ = ﬁxation index, HWE = Hardy–Weinberg equilibrium test. *P <* 0.05.
