# Supplementary material for: Genetic diversity and stock identification of small abalone (Haliotis diversicolor) in Taiwan and Japan
Source: PLoS One. 2017 Jun 29;12(6):e0179818. doi: 10.1371/journal.pone.0179818 (PMC5491045; doi:10.1371/journal.pone.0179818)
Supplement: S5 Table — (DOCX) [file pone.0179818.s005.docx]

**S5 Table.** Pairwise *F_ST_* values (below diagonal) and associated *P* values (above diagonal) based on SSRs data between populations of *Haliotis diversicolor* collected from Japan and Taiwan.

|  | JW-W | JF-W | JS-W | TE-W | TH-W | TP-C | TM-C | TE-C | TK-C |
| --- | --- | --- | --- | --- | --- | --- | --- | --- | --- |
| JW-W | － | 0.162^NS^ | 0.124^NS^ | 0.062^NS^ | 0.038* | 0*** | 0*** | 0*** | 0*** |
| JF-W | 0.031 | － | 0.729^NS^ | 0.016* | 0.002** | 0*** | 0*** | 0*** | 0*** |
| JS-W | 0.042 | 0.005 | － | 0.091^NS^ | 0.003** | 0*** | 0.002** | 0*** | 0*** |
| TE-W | 0.047 | 0.048 | 0.036 | － | 0.067^NS^ | 0*** | 0.001** | 0.002** | 0*** |
| TH-W | 0.057 | 0.070 | 0.069 | 0.037 | － | 0.123^NS^ | 0.004** | 0.004** | 0*** |
| TP-C | 0.105 | 0.116 | 0.090 | 0.094 | 0.027 | － | 0.017* | 0*** | 0*** |
| TM-C | 0.111 | 0.091 | 0.068 | 0.086 | 0.063 | 0.048 | － | 0.039* | 0*** |
| TE-C | 0.126 | 0.103 | 0.105 | 0.074 | 0.065 | 0.085 | 0.039 | － | 0*** |
| TK-C | 0.103 | 0.131 | 0.135 | 0.099 | 0.104 | 0.161 | 0.122 | 0.079 | － |

*0.05 ≥ *P* ≥ 0.01; **0.01 > *P* ≥ 0.001; ****P* < 0.001; NS, not significant.
