# Supplementary material for: Genetic diversity and stock identification of small abalone (Haliotis diversicolor) in Taiwan and Japan
Source: PLoS One. 2017 Jun 29;12(6):e0179818. doi: 10.1371/journal.pone.0179818 (PMC5491045; doi:10.1371/journal.pone.0179818)
Supplement: S6 Table — (DOCX) [file pone.0179818.s006.docx]

**S6 Table.** Hierarchical analysis of molecular variance (AMOVA) of small abalone populations based on mtDNA COI sequences collected from Japan and Taiwan by using SMOVA.

| Region groupings | | *Φ_CT_* | % variance  among groups |
| --- | --- | --- | --- |
| Among 10 Japan and Taiwan populations | |  |  |
| K= 2 | (JW-W, JF-W, JS-W); (TE-W, TH-W, TP-C, TM-C, TE-C, TK-C, TE-H) | 0.220** | 22.02 |
| K= 3 | (JW-W, JF-W, JS-W); (TE-W, TH-W, TP-C, TM-C, TE-C, TE-H); (TK-C) | 0.190** | 19.10 |
| K= 4 | (JW-W, JF-W, JS-W); (TE-W, TH-W, TP-C, TE-C, TE-H); (TM-C); (TK-C) | 0.173** | 17.31 |
| Among 7 Taiwan populations | |  |  |
| K= 2 | (TE-W, TH-W, TP-C, TE-C, TK-C, TE-H); (TM-C) | 0.049^NS^ | 4.96 |
| K= 3 | (TE-W, TH-W, TP-C, TE-C, TE-H); (TM-C); (TK-C) | 0.052* | 5.22 |
| K= 4 | (TE-W, TH-W, TP-C); (TE-C, TE-H); (TM-C); (TK-C) | 0.042^NS^ | 4.26 |

** 0.01 > *p* ≥ 0.001; * 0.05 ≥ *p* ≥ 0.01; NS, not significant.
