# Supplementary material for: Genetic diversity and stock identification of small abalone (Haliotis diversicolor) in Taiwan and Japan
Source: PLoS One. 2017 Jun 29;12(6):e0179818. doi: 10.1371/journal.pone.0179818 (PMC5491045; doi:10.1371/journal.pone.0179818)
Supplement: S7 Table — D: genetic distance (based on similarity coefficient); Shannon Information Index (I); He: expected heterozygosity (based on allele frequency); UHe: unbiased expected heterozygosity. (DOCX) [file pone.0179818.s007.docx]

**S7 Table.** **Summary of AFLP results obtained by using 6 primers in 6 populations of small abalone (*Haliotis diversicolor*).** *D*: genetic distance (based on similarity coefficient); Shannon Information Index (*I*); *He*: expected heterozygosity (based on allele frequency); U*He*: unbiased expected heterozygosity.

|  | JW-W | JF-W | JS-W | TE-C | TE-H | Total |
| --- | --- | --- | --- | --- | --- | --- |
| Sample size | 5 | 10 | 10 | 13 | 10 | 48 |
| Individually bands | 105-111 | 103-114 | 101-110 | 104-116 | 107-125 | 101-125 |
| (average) | 108 | 107.5 | 104.5 | 109.5 | 113.6 | 108.7 |
| Polymorphic bands | 70 | 68 | 58 | 59 | 71 | 125 |
| (%) | 48.61 | 47.22 | 42.65 | 42.14 | 47.65 | 73.96 |
| Private bands | 4 | 2 | 1 | 1 | 0 | － |
| Total bands | 144 | 144 | 136 | 140 | 149 | 169 |
| Band-based |  |  |  |  |  |  |
| Average *D* | 0.175 | 0.121 | 0.108 | 0.100 | 0.121 | 0.162 |
| *I* | 0.225 | 0.194 | 0.166 | 0.173 | 0.212 | 0.328 |
| SE | 0.022 | 0.020 | 0.019 | 0.020 | 0.021 | 0.019 |
| Allele frequency-based |  |  |  |  |  |  |
| *He* | 0.151 | 0.127 | 0.109 | 0.114 | 0.140 | 0.128 |
| SE | 0.015 | 0.014 | 0.013 | 0.014 | 0.014 | 0.006 |
| U*He* | 0.168 | 0.134 | 0.114 | 0.119 | 0.148 | 0.137 |
| SE | 0.017 | 0.014 | 0.014 | 0.014 | 0.015 | 0.007 |
