# Supplementary material for: Genetic diversity and stock identification of small abalone (Haliotis diversicolor) in Taiwan and Japan
Source: PLoS One. 2017 Jun 29;12(6):e0179818. doi: 10.1371/journal.pone.0179818 (PMC5491045; doi:10.1371/journal.pone.0179818)
Supplement: S1 File — (PDF) [file pone.0179818.s008.pdf]

**Table. Maximum Likelihood fits of 24 different nucleotide substitution models**

| Model | Parameters | BIC | AICc | *lnL* | (+*I*) | (+*G*) | *R* | *f*(A) | *f*(T) | *f*(C) | *f*(G) | *r*(AT) | *r*(AC) | *r*(AG) | *r*(TA) | *r*(TC) | *r*(TG) | *r*(CA) | *r*(CT) | *r*(CG) | *r*(GA) | *r*(GT) | *r*(GC) |
|---|---|---|---|---|---|---|---|---|---|---|---|---|---|---|---|---|---|---|---|---|---|---|---|
| HKY+G+I | 745 | 21714.080 | 13827.218 | -6166.714 | 0.56 | 1.36 | 5.40 | 0.272 | 0.286 | 0.255 | 0.187 | 0.022 | 0.020 | 0.158 | 0.021 | 0.215 | 0.015 | 0.021 | 0.241 | 0.015 | 0.230 | 0.022 | 0.020 |
| TN93+G+I | 746 | 21726.666 | 13829.224 | -6166.712 | 0.56 | 1.36 | 5.40 | 0.272 | 0.286 | 0.255 | 0.187 | 0.022 | 0.020 | 0.159 | 0.021 | 0.214 | 0.015 | 0.021 | 0.240 | 0.015 | 0.231 | 0.022 | 0.020 |
| T92+G+I | 743 | 21742.326 | 13876.628 | -6193.429 | 0.56 | 1.39 | 5.16 | 0.279 | 0.279 | 0.221 | 0.221 | 0.022 | 0.018 | 0.186 | 0.022 | 0.186 | 0.018 | 0.022 | 0.234 | 0.018 | 0.234 | 0.022 | 0.018 |
| GTR+G+I | 749 | 21745.931 | 13816.745 | -6157.457 | 0.56 | 1.44 | 5.38 | 0.272 | 0.286 | 0.255 | 0.187 | 0.033 | 0.025 | 0.156 | 0.032 | 0.220 | 0.009 | 0.027 | 0.247 | 0.004 | 0.227 | 0.014 | 0.005 |
| GTR+G | 748 | 21800.181 | 13881.576 | -6190.878 | n/a | 0.20 | 4.84 | 0.272 | 0.286 | 0.255 | 0.187 | 0.027 | 0.026 | 0.159 | 0.026 | 0.208 | 0.021 | 0.028 | 0.234 | 0.003 | 0.231 | 0.032 | 0.004 |
| K2+G+I | 742 | 21825.842 | 13970.725 | -6241.483 | 0.56 | 1.55 | 4.66 | 0.250 | 0.250 | 0.250 | 0.250 | 0.022 | 0.022 | 0.206 | 0.022 | 0.206 | 0.022 | 0.022 | 0.206 | 0.022 | 0.206 | 0.022 | 0.022 |
| GTR+I | 748 | 21830.645 | 13912.040 | -6206.110 | 0.59 | n/a | 4.21 | 0.272 | 0.286 | 0.255 | 0.187 | 0.043 | 0.028 | 0.165 | 0.041 | 0.194 | 0.012 | 0.030 | 0.218 | 0.004 | 0.240 | 0.018 | 0.006 |
| HKY+I | 744 | 21831.996 | 13955.716 | -6231.968 | 0.59 | n/a | 2.89 | 0.272 | 0.286 | 0.255 | 0.187 | 0.037 | 0.033 | 0.139 | 0.035 | 0.189 | 0.024 | 0.035 | 0.212 | 0.024 | 0.203 | 0.037 | 0.033 |
| TN93+I | 745 | 21853.488 | 13966.627 | -6236.418 | 0.59 | n/a | 2.86 | 0.272 | 0.286 | 0.255 | 0.187 | 0.037 | 0.033 | 0.129 | 0.035 | 0.200 | 0.024 | 0.035 | 0.225 | 0.024 | 0.187 | 0.037 | 0.033 |
| T92+I | 742 | 21855.473 | 14000.356 | -6256.298 | 0.59 | n/a | 2.90 | 0.279 | 0.279 | 0.221 | 0.221 | 0.035 | 0.028 | 0.165 | 0.035 | 0.165 | 0.028 | 0.035 | 0.208 | 0.028 | 0.208 | 0.035 | 0.028 |
| K2+G | 741 | 21868.624 | 14024.088 | -6269.169 | n/a | 0.20 | 4.82 | 0.250 | 0.250 | 0.250 | 0.250 | 0.021 | 0.021 | 0.207 | 0.021 | 0.207 | 0.021 | 0.021 | 0.207 | 0.021 | 0.207 | 0.021 | 0.021 |
| K2+I | 741 | 21907.897 | 14063.361 | -6288.806 | 0.59 | n/a | 3.73 | 0.250 | 0.250 | 0.250 | 0.250 | 0.026 | 0.026 | 0.197 | 0.026 | 0.197 | 0.026 | 0.026 | 0.197 | 0.026 | 0.197 | 0.026 | 0.026 |
| HKY+G | 744 | 21920.340 | 14044.060 | -6276.140 | n/a | 0.52 | 3.98 | 0.272 | 0.286 | 0.255 | 0.187 | 0.029 | 0.025 | 0.150 | 0.027 | 0.204 | 0.019 | 0.027 | 0.229 | 0.019 | 0.218 | 0.029 | 0.025 |
| TN93+G | 745 | 21931.583 | 14044.721 | -6275.466 | n/a | 0.52 | 4.00 | 0.272 | 0.286 | 0.255 | 0.187 | 0.029 | 0.026 | 0.140 | 0.027 | 0.215 | 0.019 | 0.027 | 0.241 | 0.019 | 0.203 | 0.029 | 0.026 |
| T92+G | 742 | 21941.815 | 14086.698 | -6299.469 | n/a | 0.52 | 3.90 | 0.279 | 0.279 | 0.221 | 0.221 | 0.028 | 0.022 | 0.176 | 0.028 | 0.176 | 0.022 | 0.028 | 0.223 | 0.022 | 0.223 | 0.028 | 0.022 |
| JC+G+I | 741 | 22696.688 | 14852.152 | -6683.201 | 0.57 | 3.16 | 0.50 | 0.250 | 0.250 | 0.250 | 0.250 | 0.083 | 0.083 | 0.083 | 0.083 | 0.083 | 0.083 | 0.083 | 0.083 | 0.083 | 0.083 | 0.083 | 0.083 |
| JC+I | 740 | 22729.363 | 14895.408 | -6705.835 | 0.59 | n/a | 0.50 | 0.250 | 0.250 | 0.250 | 0.250 | 0.083 | 0.083 | 0.083 | 0.083 | 0.083 | 0.083 | 0.083 | 0.083 | 0.083 | 0.083 | 0.083 | 0.083 |
| JC+G | 740 | 22742.683 | 14908.728 | -6712.495 | n/a | 0.23 | 0.50 | 0.250 | 0.250 | 0.250 | 0.250 | 0.083 | 0.083 | 0.083 | 0.083 | 0.083 | 0.083 | 0.083 | 0.083 | 0.083 | 0.083 | 0.083 | 0.083 |
| GTR | 747 | 23143.033 | 15235.009 | -6868.599 | n/a | n/a | 2.50 | 0.272 | 0.286 | 0.255 | 0.187 | 0.057 | 0.046 | 0.130 | 0.055 | 0.191 | 0.024 | 0.049 | 0.214 | 0.005 | 0.188 | 0.036 | 0.006 |
| T92 | 741 | 23159.767 | 15315.231 | -6914.741 | n/a | n/a | 2.90 | 0.279 | 0.279 | 0.221 | 0.221 | 0.035 | 0.028 | 0.165 | 0.035 | 0.165 | 0.028 | 0.035 | 0.208 | 0.028 | 0.208 | 0.035 | 0.028 |
| HKY | 743 | 23163.633 | 15297.935 | -6904.083 | n/a | n/a | 2.89 | 0.272 | 0.286 | 0.255 | 0.187 | 0.037 | 0.033 | 0.139 | 0.035 | 0.189 | 0.024 | 0.035 | 0.212 | 0.024 | 0.203 | 0.037 | 0.033 |
| TN93 | 744 | 23171.771 | 15295.491 | -6901.856 | n/a | n/a | 2.89 | 0.272 | 0.286 | 0.255 | 0.187 | 0.037 | 0.033 | 0.128 | 0.035 | 0.202 | 0.024 | 0.035 | 0.227 | 0.024 | 0.185 | 0.037 | 0.033 |
| K2 | 740 | 23189.456 | 15355.501 | -6935.881 | n/a | n/a | 2.88 | 0.250 | 0.250 | 0.250 | 0.250 | 0.032 | 0.032 | 0.186 | 0.032 | 0.186 | 0.032 | 0.032 | 0.186 | 0.032 | 0.186 | 0.032 | 0.032 |
| JC | 739 | 23918.491 | 16095.118 | -7306.695 | n/a | n/a | 0.50 | 0.250 | 0.250 | 0.250 | 0.250 | 0.083 | 0.083 | 0.083 | 0.083 | 0.083 | 0.083 | 0.083 | 0.083 | 0.083 | 0.083 | 0.083 | 0.083 |

NOTE.-- Models with the lowest BIC scores (Bayesian Information Criterion) are considered to describe the substitution pattern the best. For each model, AICc value (Akaike Information Criterion, corrected), Maximum Likelihood value (*lnL*), and the number of parameters (including branch lengths) are also presented [1]. Non-uniformity of evolutionary rates among sites may be modeled by using a discrete Gamma distribution (+*G*) with 5 rate categories and by assuming that a certain fraction of sites are evolutionarily invariable (+*I*). Whenever applicable, estimates of gamma shape parameter and/or the estimated fraction of invariant sites are shown. Assumed or estimated values of transition/transversion bias (*R*) are shown for each model, as well. They are followed by nucleotide frequencies (*f*) and rates of base substitutions (*r*) for each nucleotide pair. Relative values of instantaneous *r* should be considered when evaluating them. For simplicity, sum of *r* values is made equal to 1 for each model. For estimating ML values, a tree topology was automatically computed. The analysis involved 371 nucleotide sequences. Codon positions included were 1st+2nd+3rd+Noncoding. All positions with less than 95% site coverage were eliminated. That is, fewer than 5% alignment gaps, missing data, and ambiguous bases were allowed at any position. There were a total of 797 positions in the final dataset. Evolutionary analyses were conducted in MEGA6 [2].

*Abbreviations:* GTR: General Time Reversible; HKY: Hasegawa-Kishino-Yano; TN93: Tamura-Nei; T92: Tamura 3-parameter; K2: Kimura 2-parameter; JC: Jukes-Cantor.

1. Nei M. and Kumar S. (**2000**). *Molecular Evolution and Phylogenetics*. Oxford University Press, New York.
2. Tamura K., Stecher G., Peterson D., Filipski A., and Kumar S. (**2013**). MEGA6: Molecular Evolutionary Genetics Analysis version 6.0. *Molecular Biology and Evolution*30: 2725-2729.

Disclaimer: Although utmost care has been taken to ensure the correctness of the caption, the caption text is provided "as is" without any warranty of any kind. Authors advise the user to carefully check the caption prior to its use for any purpose and report any errors or problems to the authors immediately (www.megasoftware.net). In no event shall the authors and their employers be liable for any damages, including but not limited to special, consequential, or other damages. Authors specifically disclaim all other warranties expressed or implied, including but not limited to the determination of suitability of this caption text for a specific purpose, use, or application.
